# Understanding intercalative modulation of G-rich sequence folding: solution structure of a TINA-conjugated antiparallel DNA triplex

**DOI:** 10.1093/nar/gkae028

**Published:** 2024-01-28

**Authors:** Miguel Garavís, Patrick J B Edwards, Israel Serrano-Chacón, Osman Doluca, Vyacheslav V Filichev, Carlos González

**Affiliations:** Instituto de Química Física ‘Blas Cabrera’, (IQF-CSIC), Madrid 28006, Spain; School of Natural Sciences, Massey University, Palmerston North 4412, New Zealand; Instituto de Química Física ‘Blas Cabrera’, (IQF-CSIC), Madrid 28006, Spain; School of Natural Sciences, Massey University, Palmerston North 4412, New Zealand; School of Natural Sciences, Massey University, Palmerston North 4412, New Zealand; Instituto de Química Física ‘Blas Cabrera’, (IQF-CSIC), Madrid 28006, Spain

## Abstract

We present here the high-resolution structure of an antiparallel DNA triplex in which a monomer of *para*-twisted intercalating nucleic acid (*para*-TINA: (*R*)-1-*O*-[4-(1-pyrenylethynyl)phenylmethyl]glycerol) is covalently inserted as a bulge in the third strand of the triplex. TINA is a potent modulator of the hybridization properties of DNA sequences with extremely useful properties when conjugated in G-rich oligonucleotides. The insertion of *para*-TINA between two guanines of the triplex imparts a high thermal stabilization (Δ*T*_M_ = 9ºC) to the structure and enhances the quality of NMR spectra by increasing the chemical shift dispersion of proton signals near the TINA location. The structural determination reveals that TINA intercalates between two consecutive triads, causing only local distortions in the structure. The two aromatic moieties of TINA are nearly coplanar, with the phenyl ring intercalating between the flanking guanine bases in the sequence, and the pyrene moiety situated between the Watson–Crick base pair of the two first strands. The precise position of TINA within the triplex structure reveals key TINA–DNA interactions, which explains the high stabilization observed and will aid in the design of new and more efficient binders to DNA.

## Graphical Abstract

**Graphical Abstract:**
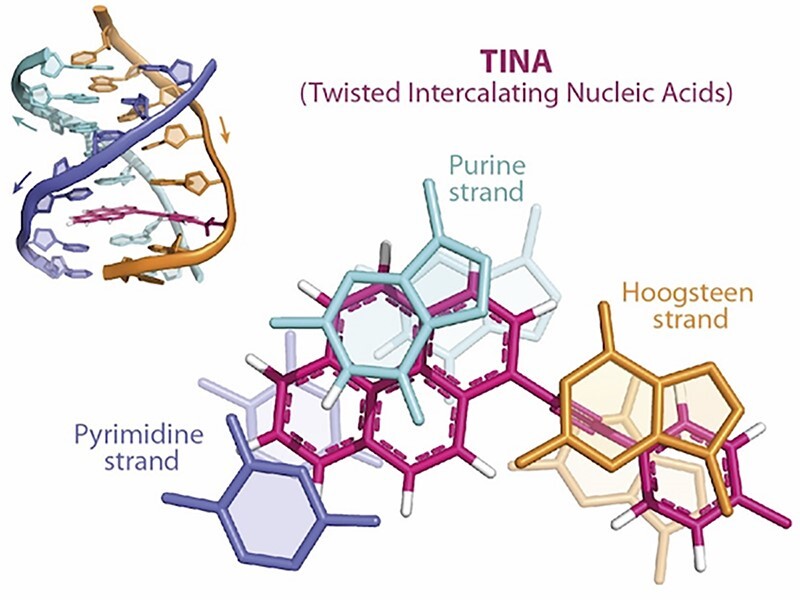

## Introduction

Triplex DNA is formed by Hoogsteen base-pairing of a third strand to the purine tract of a DNA duplex (1–4). In the last years, triplex DNA have been detected inside cells through in-cell NMR (5) and using specific triplex antibodies (6–9). This, together with the identification of proteins that bind triplex (10–12), suggests that its formation occurs *in vivo* and has biological significance. The overrepresentation of putative triplex forming sequences in regulatory regions of the genome (13–15) suggests that triplex DNA structures may play roles in gene expression and DNA metabolism. Additionally, triplex DNA formation has been associated with double strand breaks (DSB) occurring during replication (15,16), which has attracted the interest on these structures as possible tools for novel gene editing technologies such as CRISPR-Cas (17–19).

The formation of a DNA triplex by using exogenous triplex-forming oligonucleotides (TFO) targeting specific regions of a gene has been widely used as a potent strategy to modulate gene expression (4,20–25) and to direct or induce site-specific effects such us damage, mutagenesis or genetic instability on targeted DNA sequences (4,26–28). Interestingly, most of the annotated human genes contain at least one unique poly-purine sequence that could serve as TFO target site (29). On the other hand, triplex formation constitutes the basis of novel nanotechnological devices with multiple applications (30).

**Figure 1.**
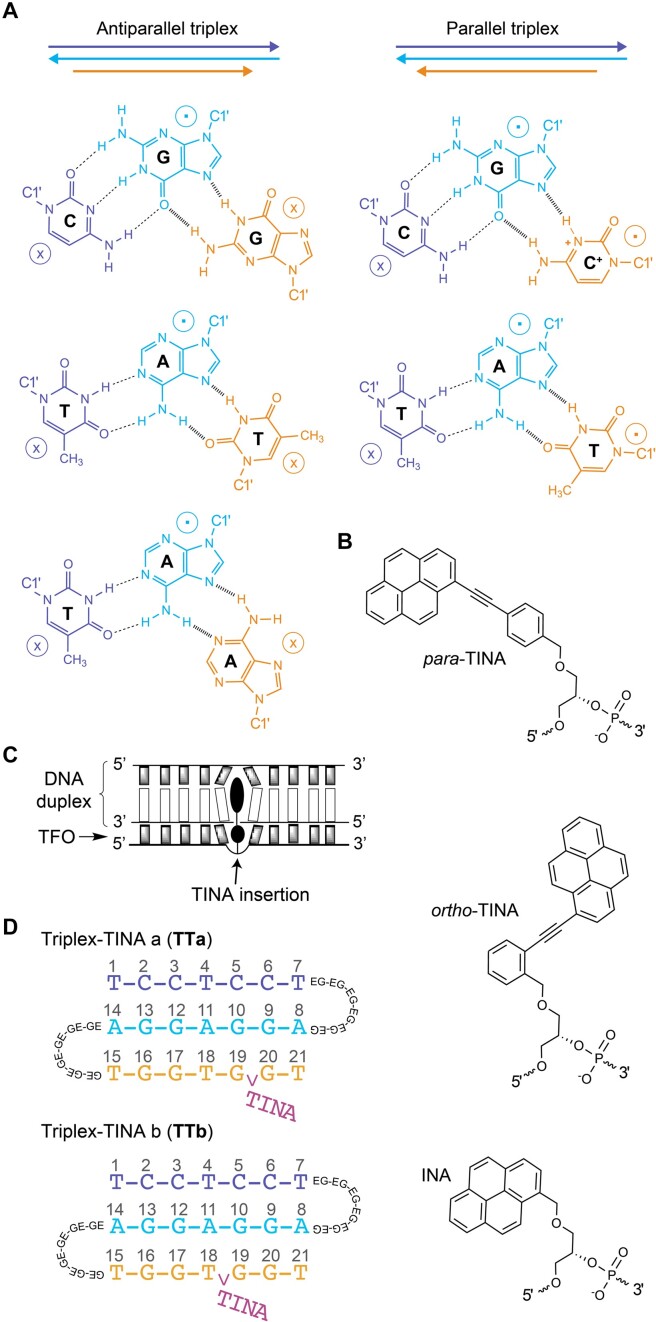
(**A**) Strand orientation and triads in antiparallel (left) and parallel (right) triplexes: the third strand (yellow) binds to the homopurine sequence (cyan) of a duplex through Hoogsteen or reversed Hoogsteen base pairs (hashed bonds). The two orientations of phosphodiester backbones are indicated by the symbols ‘⊗’ and ‘⊙’. (**B**) Chemical structure of *para*-TINA, *ortho*-TINA and INA. (**C**) Scheme showing the bulge insertion of TINA into the TFO part of the triplex. (**D**) Scheme of the modified antiparallel triplexes TTa (top) and TTb (bottom) showing three DNA strands linked by two loops of six ethylene glycol (EG) units and the TINA monomer conjugated between the residues G19 and G20 (TTa) and T18 and G19 (TTb).

Parallel triplex formation by using pyrimidine-rich TFOs can be limited at physiological pH as protonation of cytosines -and thus acidic pH- is required to form Hoogsteen base pairs with the guanines of the target duplex (31) (Figure 1A). On the contrary, guanine-rich TFOs, which form antiparallel triplexes appear as more adequate TFO candidates as they are insensitive to pH (Figure 1A). However, the tendency of these sequences to self-fold into G-quadruplex structures in the presence of monovalent cations (K^+^ and Na^+^) present in the cell context, can completely abolish triplex formation (32,33).

Several strategies have been devised to overcome these limitation (31,34). One approach involves the use of modulators that alter the hybridisation properties of DNA sequences, such as intercalating nucleic acids (Figure 1B). Within this category, the *para*-twisted intercalating nucleic acid monomer (*para*-TINA: (*R*)-1-*O*-[4-(1-pyrenylethynyl)phenylmethyl]glycerol) (Figure 1B) inserted as a bulge into the TFO (Figure 1C), has demonstrated remarkable efficacy in circumventing guanine-mediated self-association of G-rich TFOs, particularly when positioned in the middle of the G-tract (35,36). In addition, for cellular applications it is important that complexes of TINA-containing TFOs with complementary RNAs were significantly destabilised, which makes TINA–TFOs useful molecules targeting genomic DNA (37,38) and not RNA. TINA was designed to provide stabilization to the triplex through stacking interactions with duplex and TFO bases simultaneously. Thus, it was conceived containing two aromatic platforms; a pyrene ring and a phenyl ring aimed to intercalate between two consecutive duplex base pairs and TFO bases, respectively. Additionally, the presence of a linear triple bond connection between both rings provides rotational flexibility allowing the aromatic rings to be in different planes (38). Several studies have shown that TINA increases thermodynamic stability of both parallel (38,39) and antiparallel (35,36) DNA triplexes without compromising sequence-specificity, which agrees with other pyrene-containing DNA probes (40,41). Importantly, TINA-containing TFOs have been proven efficient in regulation of gene expression (36). In contrast, *ortho*-TINA (Figure 1B) in which 1-ethynylpyrene is attached to the *ortho* position of the benzene ring stabilized Watson–Crick-type duplexes and had minimal influence on parallel triplexes (42). The behaviour of *ortho*-TINA was mimicking original intercalating nucleic acids (INA) which stabilized antiparallel duplexes (43) but destabilized triplexes (44). Although some models of *para*-TINA intercalation in duplex and parallel triplex have been published, no high-resolution studies have been reported so far. Here, we use NMR methods to determine the detailed three-dimensional structure of an antiparallel intramolecular triplex with a single insertion of a *para*-TINA monomer in the Hoogsteen strand (Figure 1D). Since the structural information on triplex is very limited, we focused on one of the few sequences whose structure has been determined (45). To facilitate the assignment of the NMR spectra, the original five-thymine loops were substituted by six ethylene glycol (EG6) linkers (Figure 1D).

## Material and methods

### Oligonucleotide synthesis and purification

Oligonucleotide synthesis was performed on a Mer-Made-4 automated DNA synthesizer under conditions described previously (35).

### CD and UV experiments

Circular dichroism (CD) spectra were recorded on a Jasco J-815 spectropolarimeter. UV spectra were recorded on a Jasco V-730 spectrophotometer. Both instruments are fitted with Peltier thermoelectric cooling modules. Experiments were conducted in a buffer containing 25 mM sodium phosphate and 100 mM NaCl at pH 7. Samples were initially heated at 90°C for 5 min and slowly allowed to cool to room temperature and stored at 4°C until use. Samples were placed in quartz cuvettes with 1 cm pathlength. UV melting curves were recorded at the wavelength of 256 nm, with a heating rate of 0.5°C·min^−1^. Uncertainties in *T*_M_ values are estimated to be ±0.5°C. CD spectra were recorded at various temperatures after 5 min equilibration. Thermodynamic analysis of UV-melting curves was performed according to the published protocol (46) assuming intramolecular equilibrium which is supported by the presence of isosbestic points in CD spectra at various temperatures. Melting point was determined as a temperature in which half of the complex was melted. Complete details regarding the determination of thermodynamic parameters from UV-melting profiles are provided in the Supplementary Data.

### NMR experiments

Samples for NMR experiments were suspended in 200 μl of either D_2_O or H_2_O/D_2_O 9:1 in a buffer containing 25 mM sodium phosphate and 100 mM NaCl, pH 7. Final oligonucleotide concentration was around 0.5 mM. NMR spectra were acquired on Bruker Avance spectrometers equipped with cryoprobes and operating at 600 or 800 MHz. NMR data was processed with Bruker Topspin software. TOCSY spectra were recorded with the standard MLEV17 spinlock sequence and with an 80 ms mixing time. NOESY spectra in H_2_O were acquired with mixing times ranging from 50 to 250 ms. Water suppression was achieved by including a WATERGATE (47) module in the pulse sequence prior to acquisition. Two-dimensional experiments were carried out at temperatures ranging from 5 to 25°C. The spectral analysis program NMRFAM-SPARKY (48) was used for peak assignment and semiquantitative NOE analysis.

### Experimental NMR constraints

Distance constraints were obtained from an estimation of NOE intensities. In addition to these experimentally derived constraints, hydrogen bond and planarity constraints for the base pairs were used in the initial DYANA (49) calculations. Target values for distances and angles related to hydrogen bonds were set to values obtained from crystallographic data in related structures (50). Due to the relatively broad line-widths of the sugar proton signals, J-coupling constants were not accurately measured, but only roughly estimated from DQF-COSY cross-peaks. Loose values were set for the sugar dihedral angles ν_0_, ν_1_, ν_2_, ν_3_ and ν_4_ to constrain the 2′-deoxyribose conformation to the south domain. No backbone angle constraints were employed. Distance constraints with their corresponding error bounds were incorporated into the AMBER potential energy by defining a flat-well potential term.

### Structural determination

Structures were calculated with the program DYANA (49) and further refined with the SANDER module of the molecular dynamics package AMBER 18.0 (51). The resulting DYANA structures were used as starting points for the AMBER refinement. The refined structures were first placed in a truncated octahedral box of water molecules with minimal distance between the solute and the box border of 12 Å using the TIP3P model. Eighteen sodium ions were added to neutralize the total charge of the system. The BSC1 force field (52) and suitable parameters for TINA were used to describe the modified oligonucleotide. The specific protocols used for AMBER refinement have been described in detail elsewhere (53). Here, 25 ns restrained molecular dynamics (rMD) were run using experimental NMR constraints involving non-exchangeable protons as well as distance and angle constraints for triads formation (triad constraints). Final structures were obtained extracting ten structures from the rMD trajectories and further relaxation of the structures removing the triad constraints and using the same NMR constraints used during rMD simulations plus NMR constraints involving interchangeable protons. Analysis of the representative structures as well as the MD trajectories was carried out with the programs MOLMOL (54), X3DNA (55), CURVES (56) and PyMOL (57). Energy decomposition of MD trajectories was performed using the Gromacs-based utility Gromologist (58,59).

Unbiased molecular dynamics calculation without experimental constraints were performed to evaluate the stability and consistency of the NMR structures. These runs consisted of three replicas of a 100 ns simulation starting form a structure derived from NMR data (rMD calculations).

Partial atomic charges for TINA were calculated using the RESP model (60) after geometry optimization of the residue having its 5′ and 3′ oxygens capped with hydrogens. The electrostatic potential energy were carried out at the Hartree–Fock level of theory using the 6–31G(d) basis set for consistency with other atomic charges in the AMBER force field (61). For comparison purposes, the partial atomic charges of TINA were also calculated using the B3LYP/6–31G(d,p) method. Values and differences between charges obtained using HF and B3LYP are shown in Supplementary Table S9. Additional force field parameters needed were obtained from BSC1 (62) or GAFF (63).

## Results

### TINA insertion increases the thermal stability of the antiparallel triplex

We synthesized the DNA–TINA conjugates TTa and TTb, containing three oligonucleotide tracts of seven residues separated by two EG6 linkers. The oligonucleotide sequences were designed to fold as antiparallel triplexes with the EG6 linkers in the loops (Figure 1D). A TINA monomer was conjugated between the last two guanines (G19 and G20) of TTa and between T18 and G19 of TTb (Figure 1D). According to Doluca *et al.* (35), the affinity constant of TINA-containing oligonucleotides forming antiparallel triplex is highest when TINA is flanked by two purine or two pyrimidine residues. Thus, location of TINA within TTa was conceived to favour a more stable antiparallel triplex compared to TTb.

We used UV melting curves and CD and ^1^H-NMR spectroscopy to evaluate the thermal stability of these constructs, as well as the unmodified control triplex (UT). UV melting curves of the unmodified and TINA-modified triplexes are shown in Supplementary Figure S1, confirming the strong thermal stabilization induced by TINA with *T*_M_ values of 69ºC and 78ºC for UT and TTa, respectively. The TTb triplex showed several melting transitions indicative of suboptimal position of TINA in the triplex. Presence of isosbestic points in CD spectra of TTa at various temperatures (Supplementary Figure S1) confirms melting of the complex at equilibria. This gives the same *T*_M_ value as determined by UV melting, which is expected for melting of unimolecular triplex at different concentrations, i.e. UV melting was performed at 2 μM and CD melting at 20 μM. Considering a unimolecular reversible melting transition, thermodynamic data analysis of the melting curves was conducted, as described in the Supplementary Data. This analysis revealed that triplex stabilisation by TINA (ΔΔ*G_298_* = −9.7 kJ/mol) is attributed to the large enthalpic contribution (ΔΔ*H* = −41 kJ/mol) that is partially deprived by the unfavourable entropic component (Δ(TΔ*S*) = –31.3 kJ/mol), if comparing data of TTa and UT triplexes.

**Figure 2.**
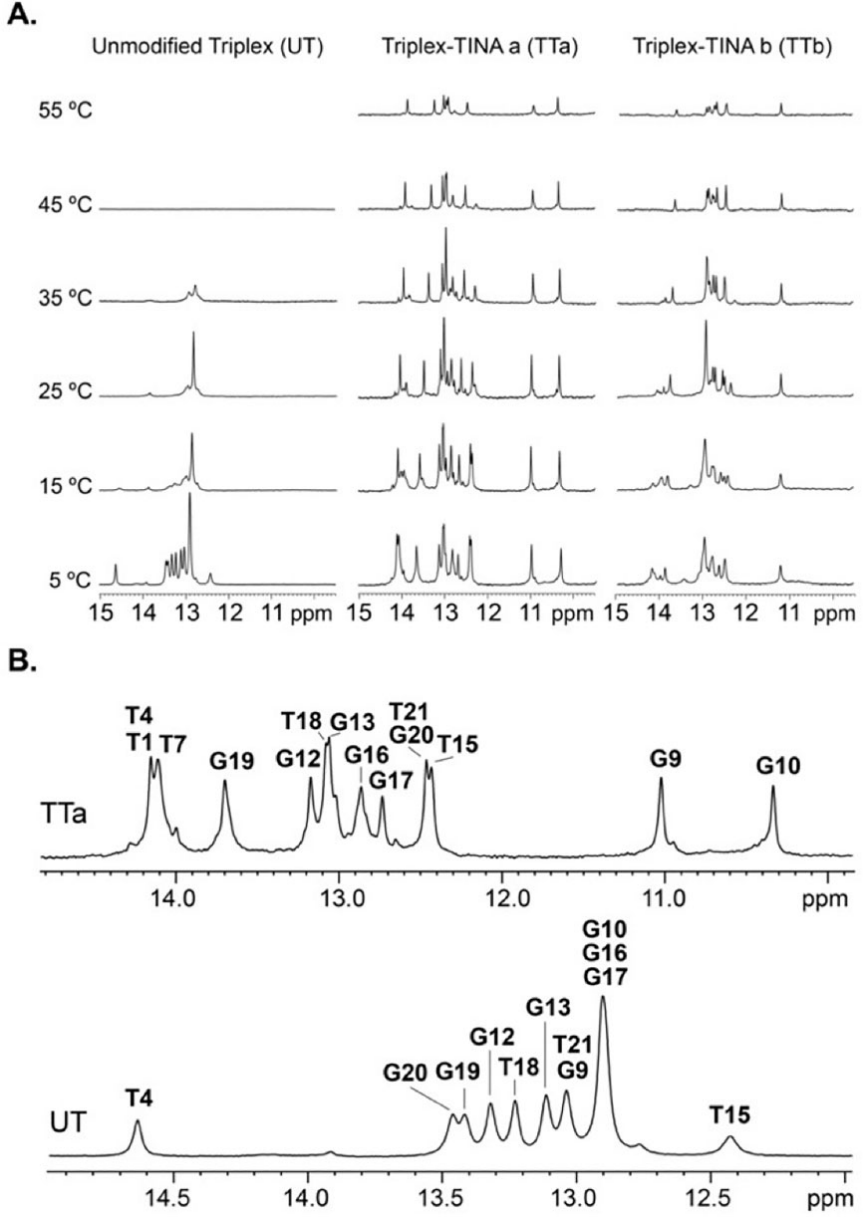
(**A**) Exchangeable proton regions of the 1D ^1^H-NMR spectra of UT (left), TTa (middle) and TTb (right) at 400 μM acquired at different temperatures. (**B**) Expanded view of the imino region of UT and TTa at 5ºC showing the assignment of the imino protons. Buffer conditions: 25 mM sodium phosphate, 100 mM NaCl, pH 7, H_2_O/D_2_O 90:10.

The 1D NMR spectra at 5ºC (Figure 2) show sharp signals in the range of 12–15 ppm, characteristic of imino protons involved in Watson–Crick and Hoogsteen base pairs. Thermal denaturation of UT and TTa was also followed by recording ^1^H-NMR spectra at different temperatures and observing the decay of imino signal intensities as temperature increases. Whereas imino signals in UT completely disappear at 45ºC, in TTa they are still observed at 55ºC (Figure 2), indicating that the TTa triplex remains folded at much higher temperature than UT. In order to determine the structural reasons behind this remarkable thermal stabilization induced by TINA, we conducted the structural determination of TTa at atomic resolution.

### Assignment of NMR spectra

**Figure 3.**
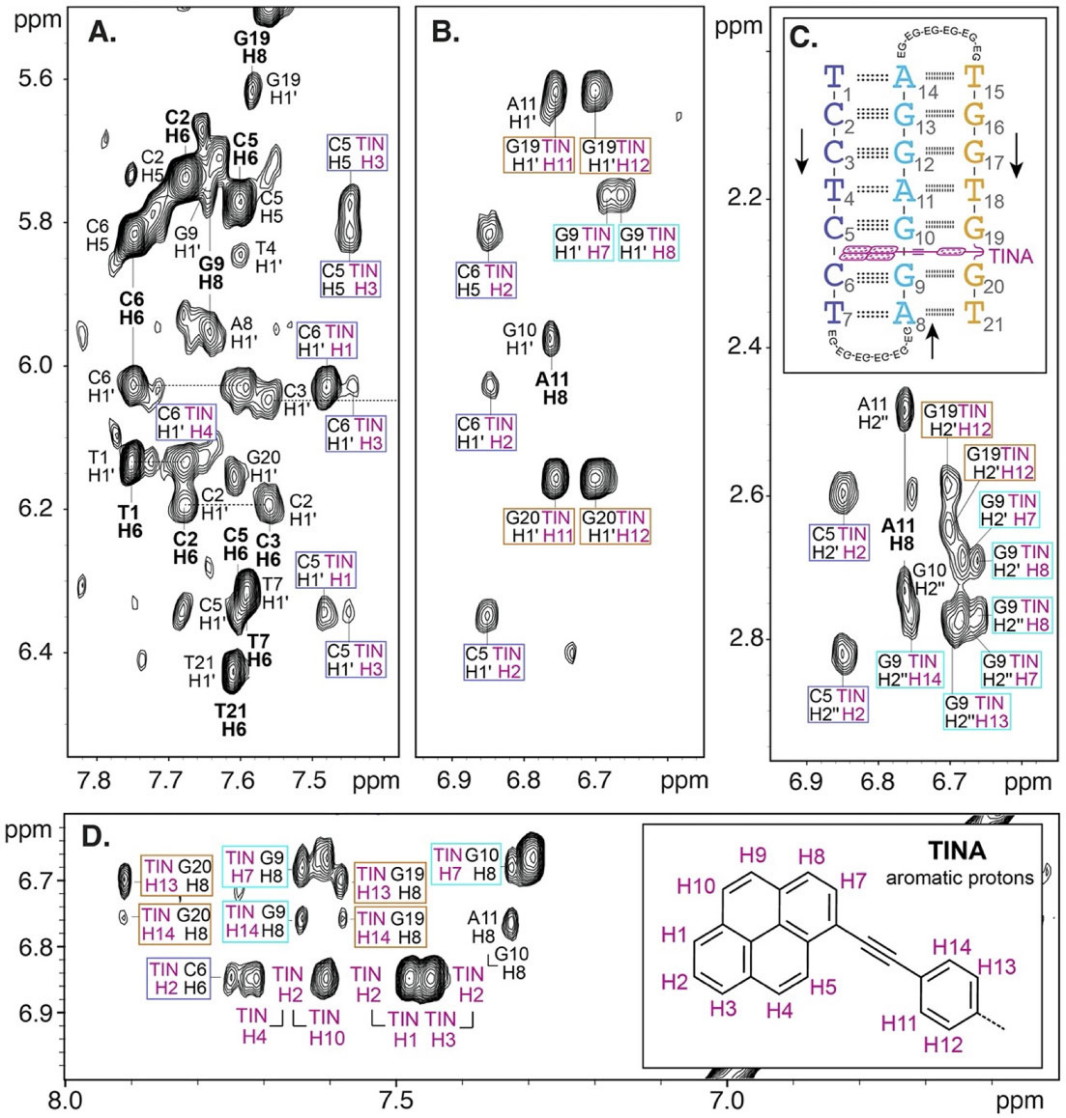
Different regions of the NOESY spectra of TTa in D_2_O at 5 ºC showing sequential assignment of first DNA strand of TTa (panel A, dashed lines) and cross-peaks between TINA protons and: (**A** and **B**) H5 of C5 and C6 and H1’ protons of C5, C6, G9, G19 and G20; (**C**) H2’ and H2’’ of C5, G9 and G19; and (**D**) aromatic protons of C6, G9, G10, G19 and G20. Label frames are coloured in blue slate (C5 and C6), cyan (G9 and G10) and orange (G19 and G20). Aromatic protons of the nucleotides are labelled in bold. Some aromatic-aromatic cross-peaks of neighbour aromatic TINA protons are also shown (magenta interconnected labels in panel D). A scheme of the antiparallel triplex and the chemical structure of TINA with labelled aromatic hydrogens are shown in the inset of panels C and D, respectively.

Assignment of non-exchangeable proton signals of TTa was performed using NOESY and TOCSY spectra acquired in D_2_O. In general, due to the presence of long poly-purine tracks, NMR spectra of antiparallel triplexes are poorly dispersed and difficult to assign. However, the presence of TINA provokes a significant dispersion of the NMR signals, facilitating their assignments. This signal dispersion effect has also been observed in G-quadruplex structures having substitutions of the pyrene-containing nucleobase U^Py^ (5-(pyren-1-yl-ethynyl)-dUMP) (64). Connectivity between consecutive residues along the first strand can be followed from T1 to C5 through H2′/H2″(i) – H6 (i + 1) and H1′(i) – H6(i + 1) cross-peaks (Figure 3A). The lack of sequential cross-peaks between C5 and C6 is consistent with the intercalation of TINA. Connectivity is again recovered between C6 and terminal T7 (Figure 3A). Further sequential cross-peaks of medium intensity along the first strand such as H6(i)–CH_3_(i + 1) in steps C–T (Supplementary Figure S2) and H6(i)–H5(i + 1) in steps C2–C3 and T4–C5 are observed and indicative of a right-handed helix formation.

Sequential assignment along the second strand starts by identifying the H8 aromatic proton of A8, which shows cross-peaks between 3.0 and 3.6 ppm with methylene protons of the first EG6 loop. Weak inter-residual cross-peaks lead to the identification of G9H8. The connectivity is lost between G9 and G10, supporting also the intercalation of TINA between these residues of the second strand. G10 is identified by the characteristic NOE between its aromatic proton and the methyl protons of the thymine in the next triad (T18) (Supplementary Figure S2). The analogous NOE between G13H8 and T15CH_3_ is observed for the other G-A step in the second strand (Supplementary Figure S2). From G10, the connectivity is maintained along the rest of the residues in the strand. As observed for T7H6 and A8H8, A14H8 also shows cross-peaks with protons of EG units linking the second and the third strand. The H2 protons of adenines were identified by their cross-peaks with imino protons of the first strand (Supplementary Figure S3) and with EG6 protons of the first and second EG6 linkers in the case of the terminal adenines A8 and A14, respectively.

The aromatic proton of T15 shows cross-peaks with protons of the second EG6 loop and a particularly intense intra-residual cross-peak with H2′ which appears rather shifted to high-field (Supplementary Figure S2). The connectivity between T15 and G16 is detected through a weak sugar-base T15H2′–G16H8 cross-peak (Supplementary Figure S2) and aromatic-aromatic NOE T15H6-G16H8 (Supplementary Figure S3). Sugar-base connectivity is lost between G16 and G17 and appears again between G17 and T18. The notably intense G17H8-T18CH_3_ (Supplementary Figure S2) is evidential of the right-handed helical nature of the third strand. The analogous NOE in the other G–T step, G20H8–T21CH_3_, is also detected (Supplementary Figure S2). Likewise, moderately intense cross-strand NOEs G10H8–T18CH_3_ and G13H8–T15CH_3_ (Supplementary Figure S2) confirm the reverse-Hoogsteen base-pairing between adenines and thymines in the second and third strand, respectively (Figure 1). As previously observed for the steps C5–C6 and G9–G10, the connectivity is also lost between G19 and G20 which is consistent with the intercalation of TINA between the triads C5:G10*G19 and C6:G9*G20. Finally, G20 also shows sugar-base connections with the last residue of the triplex, T21.

The exchangeable signals observed between 12 and 15 ppm in the NOESY spectra in H_2_O were assigned to imino protons involved in the formation of all the seven triads of the triplex. Imino protons of thymines of the first strand were identified by their intense cross-peaks with the H2 of their Watson–Crick-paired adenines (Supplementary Figure S4). In the case of the internal A11:T4 base pair, the cross peaks between T4H3 and A11 amino protons are also detected (Supplementary Figure S4). Imino protons of the second strand (G9H1, G10H1, G12H1 and G13H1) were assigned attending to their strong NOEs with amino protons of their base-paired cytosines of the first strand (C6, C5, C3 and C2, respectively) (Supplementary Figures S4 and S5). Interestingly, the imino protons of G9 and G10 resonate and are very shifted to high field with respect to the rest of imino protons (Supplementary Figure S5). Finally, the imino protons of thymines and guanines of the third strand show the characteristic strong NOEs with the H8 aromatic protons of their Hoogsteen base-paired purines of the second strand (Supplementary Figure S4). Moreover, imino protons of these residues exhibit cross-peaks of weak or medium intensity with H2′, H2″ and H8 protons of the nucleotide preceding their base-paired partner in the second strand (Supplementary Figures S4 and S6). The chemical shifts of all the DNA assigned protons are shown in Supplementary Table S1.

NMR signals of TINA aromatic protons (TIN H) were assigned by analysing TOCSY and NOESY spectra in D_2_O (Supplementary Figure S3, Supplementary Table S2). Identification of TIN H2 proton was unambiguous due to its intense cross-peaks with TIN H1 and TIN H3 observed in both NOESY and TOCSY spectra. Consequently, TIN H3 shows two cross-peaks in the NOESY that were assigned according to their intensity as TIN H4 (stronger) and TIN H5 (weaker) which show, in turn, intense TOCSY and NOESY cross-peaks with each other. TIN H5 is further correlated in the NOESY to the proton of the phenyl ring TIN H11. On the other hand, TIN H1 shows two NOEs, the stronger of them with TIN H10 and the weaker with TIN H9. The two later protons are corelated in the NOESY and TOCSY spectra. TIN H9 shows an intense cross-peak with TIN H8, which in turn resonates very close to its neighbour TIN H7, so the NOE and TOCSY cross-peaks correlating them lay very close to the diagonal of the spectra. The protons of the phenyl ring TIN H11 and TIN H12 resonate at the same chemical shift and are correlated in the NOESY and TOCSY (peaks close to the diagonal) with TIN H13 and TIN H14 which are also degenerated.

### NOESY spectra exhibit numerous short distances between TINA and DNA protons

A total of 45 cross-peaks between DNA and TINA protons were identified (Supplementary Table S3). All the residues of the triads flanking the intercalated TINA (C5:G10*G19 and C6:G9*G20) show contacts with TINA protons. The precise position of TINA between the triads is defined by the presence and intensity of NOEs between TINA protons and DNA protons of the six surrounding nucleotides. On the one hand, several NOEs correlating sugar protons of C5 and C6 with the TINA protons H1, H2, H3, H4 and H10 (Figure 3A–C) strongly constraint the position of the TINA pyrene ring. Additionally, the H5 of C5 and C6 shows moderately intense cross-peaks with H2 and H3 of TINA (Figure 3A and B) which further inform about the position of that side of the pyrene ring. Consistently, sugar protons of G9 show NOEs with the pyrene ring TINA protons H7 and H8 (Figure 3B and C). The aromatic protons of G9 and G10 also show relatively intense cross-peaks with TINA H8 (Figure 3D). The protons of the TINA phenyl ring correlate with protons of the flanking residues in the sequence G19 and G20. The particularly intense NOEs between H1′ of the two guanines and TINA H11 and H12 (Figure 3B) are indicative of the penetration degree of TINA into the triplex. The position of the phenyl ring is further determined by NOEs correlating the aromatic protons of G19 and G20 with the other two protons of the ring H13 and H14 (Figure 3D).

The unmodified version of the same sequence (UT) was also studied by two-dimensional NMR. Most of the chemical shifts of UT protons (Supplementary Table S4) are similar to the ones assigned for TTa (Supplementary Table S1). However, the presence of TINA within the sequence affects remarkably the chemical shift of some protons. Consistently with the position of TINA, the highest chemical shift differences between UT and TTa protons corresponds to those of C5:G10*G19 and C6:G9*G20 triads (Supplementary Table S5 and Supplementary Figure S7) some of which differ in more than 1 (C5H41, C6H41 and G20H1) and 2 ppm (G9H1 and G10H1).

### Structural features of TTa

**Figure 4.**
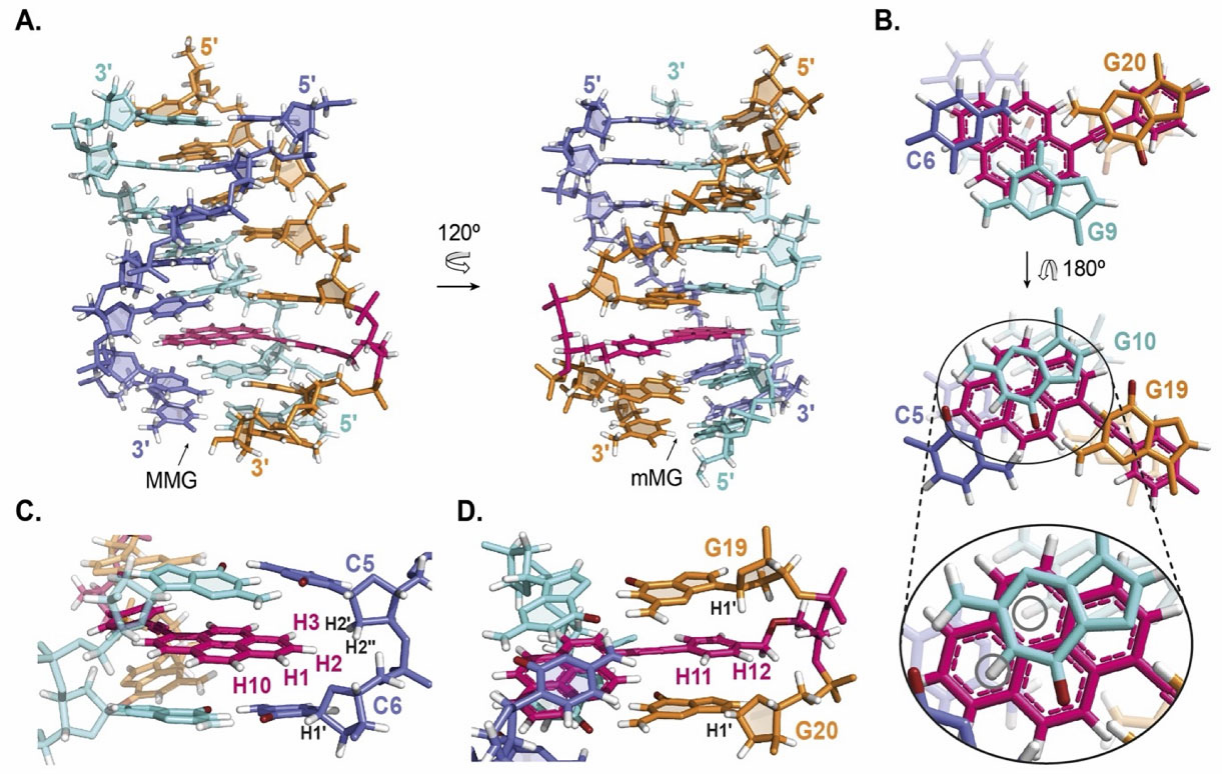
(**A**) Structure of TTa viewed from the major-major groove (MMG), and minor-major groove (mMG) perspectives. (**B**) Detailed view of the triads C6:G9*G20 (top) C5:G10*G19 (middle) showing the stacking of TINA aromatic rings on the flanking triads. Expanded view of G10 laying above the pyrene ring of TINA (B bottom) showing the position of G9 and G10 imino protons (inside grey circles) under the influence of the pyrene ring of TINA. (**C**) Detailed lateral view from the minor groove (mG) illustrating the close position of TINA H1, H2, H3 and H10 protons to sugar protons of C5 and C6. (**D**) Detailed lateral view from the MMG groove showing the proximity of TINA H11 and H12 to the H1’ of their flanking guanines G19 and G20.

On the basis of the experimental constraints derived from NMR spectra (Supplementary Table S6), we carried out the structural determination of TTa by restrained molecular dynamics methods (rMD). Two different views of the structure (PDB ID: 8PWR) are shown in Figure 4A. The structures were calculated as a trimolecular complex since the EG6 connectors were not assigned in the NMR spectra and, therefore, were not considered in the calculation. All final structures exhibit reasonably low energy values and no average distance violation larger than 0.25 Å (Supplementary Table S6). All the residues are very well defined with overall RMSD values around 0.6 Å (Supplementary Table S6). The terminal residues are equally well-defined as the rest of the structure (Supplementary Figure S8). The consistency of the resulting structures was further verified by running longer unbiased molecular dynamics trajectories. The analysis of RMSD variation along three 100 ns trajectories exhibit good convergence for both the overall structures and the TINA interaction site, with RMSD values sustained around 2 Å along the trajectory (Supplementary Figure S9).

The structure of TTa (Figure 4A) is a right-handed triple helix with the geometrical parameters shown in Supplementary Tables S7 and S8. Sugar puckers of most residues are in the general south domain, except T18, C5, T15 and G20 that are in the east domain, and G19 that adopts north or south puckering in different structures (Supplementary Table S7). Glycosidic angles of most nucleotides are *anti* with χ values in the range of −110º to −150º except for G9, G10, G16, T18 and G19 that adopt a high *anti* glycosidic conformation (χ values from −50º to −103º) (Supplementary Table S8). General helical parameters (see Supplementary Figure S10) are consistent with expected values for an antiparallel triplex, except in the TINA insertion region, where notably high rise and reduced twist values are observed.

The structure is stabilized by three T:A*T and four C:G*G (Figure 1A) triads and a TINA monomer intercalated between the triads C5:G10*G19 and C6:G9*G20. The phenyl and the pyrene rings of TINA exhibit a slight tilt with respect to each other while remaining equidistant to the two triads (Figure 4A). The pyrene ring lays between the Watson–Crick base-pairs C5:G10 and C6:G9 (Figure 4B) while the phenyl ring intercalates between the G19 and G20 bases, exhibiting a larger stacking with the former (Figure 4B).

A detailed view of the structure confirms all the DNA-TINA contacts observed in the NOESY spectra. Thus, TINA H1, H2, H3 and H10 are close to the residues of the first strand (C5 and C6) (Figure 4C); TINA protons H7 and H8 are near bases of the second strand (G9 and G10); and protons H11 and H12 of the phenyl ring are at close distance to residues of the third strand (G19 and G20) (Figure 4D). This TINA location explains the changes in chemical shifts with respect to the unmodified triplex (Supplementary Figure S7). Especially evident are the highly shifted imino signals of G9 and G10, observed between 10 and 11 ppm in TTa (Figure 2 and Supplementary Figure S5) and at 13.05 ppm and 12.92 ppm in UT. These protons are notably influenced by ring-current effects, owing to their close proximity to the TINA pyrene ring, as shown in Figure 4B. The same effect is observed in the NMR spectra of the sequence TTb, where TINA is conjugated between T18 and G19 instead of G19 and G20. In this case TINA intercalates between the T4:A11*T18 and C5:G10*G19 triads so the pyrene ring only stands above one imino proton of the second strand (G10H1). Indeed, only one signal is observed between 10 and 12 ppm which presumably corresponds to G10H1 (Figure 2). This characteristic chemical shift of the guanine imino protons flanking TINA in the purine strand could be used as a sign to confirm TINA–TFOs binding by NMR.

## Discussion

Triplex formation is a promising tool for selectively influencing gene expression (2,65). Antiparallel triplexes offer several advantages over parallel ones because of their stability under physiological conditions. Despite this, there is a striking scarcity of structural information regarding this specific triplex conformation. To date, only one high-resolution structure of an antiparallel DNA triplex is available in the PDB, and it was reported 30 years ago (45). In the present work we demonstrate that a single insertion of TINA is enough to significantly increase the thermal stability of an antiparallel triplex in comparison to its unmodified analogue. We determine the structure of the modified triplex in solution, which represents the first high-resolution structure of a TINA-containing DNA molecule and unveils the binding mode of this modification and the structural reasons behind its stabilization effect.

Our structural determination shows that the position of TINA between triads mostly reproduces the one anticipated by its rational design, with the pyrene ring stacking on the base pairs of the duplex and the phenyl ring sandwiched by the flanking bases attached to it. As was predicted by molecular modelling and fluorescence experiments on a similar antiparallel triplex (36), the location of the pyrene ring is closer to the duplex moiety. The structure shows that, specifically, the pyrene ring stacking occurs on the bases of the purine strand G9 and mostly G10 (Figure 4B). Given so, the use of polyaromatic platforms larger than pyrene may also fit well between triads and might further enhance the stability of the triplex through stacking interactions covering more surface of the duplex base pairs. Likewise, some analogous substituents of the phenyl ring would enlarge the stacking on the contiguous bases of the third strand. In this regard, Bomholt et al. (66) reported that the *T*_M_ of a parallel triplex conjugated with a TINA having a naphthalene ring instead of a phenyl ring was 2ºC higher than the one of the same triplex modified with a TINA having a phenyl ring. By the virtue of the chemical design, *ortho*-TINA and particularly INA (Figure 1B) cannot position pyrene optimally for intercalation between the duplex part of the triplex as *para*-TINA does, thus explaining contrasting properties of these molecules.

The triple bond of TINA was conceived to allow the fitting of the two aromatic platforms on bases having different tilt. Indeed, the bases of the Hoogsteen strand run coplanar along the strand but in a different plane with respect to the base pairs of the duplex moiety. The structure shows that, both rings of TINA are slightly twisted to each other. The torsion angle between both aromatic planes ranges from 10º to 20º depending on the refined structure analysed being the average torsion angle 16.5º. This data is in good agreement with the molecular modelling results obtained for both antiparallel (36) and parallel (38) triplexes that reported torsion angles of 1–10º and 15.3º, respectively. A greater torsion angle might be deleterious for triplex stabilisation as previously observed for the triazole-TINA in which 1,2,3-triazole was used instead of the triple bond in *para*-TINA imposing a torsion angle of 35–40º and a significantly lower level of parallel triplex stability (67).

The analysis of melting curves for TTa and UT triplexes clearly indicates that TINA stabilization is of enthalpic origin. The insertion of TINA into the triplex introduces not only the pyrene intercalator but also a bulge in the structure and an additional negatively charged phosphate. These structural features must be accommodated within the triplex, introducing an increased unfavorable entropy component to the free Gibbs energy of triplex formation. TINA intercalation within the triplex structure suggests that the stabilizing effect is primarily due to stacking interactions. The energy decomposition of our MD data enables the quantification of the TINA effect in the triplex, yielding values of –181.6 kJ/mol and –258.3 kJ/mol for the coulombic and Lennard-Jones terms, respectively. These values are sufficiently large to compensate for the distortions in the triplex structures required for the formation of the intercalation site and any associated negative entropic effect.

**Figure 5.**
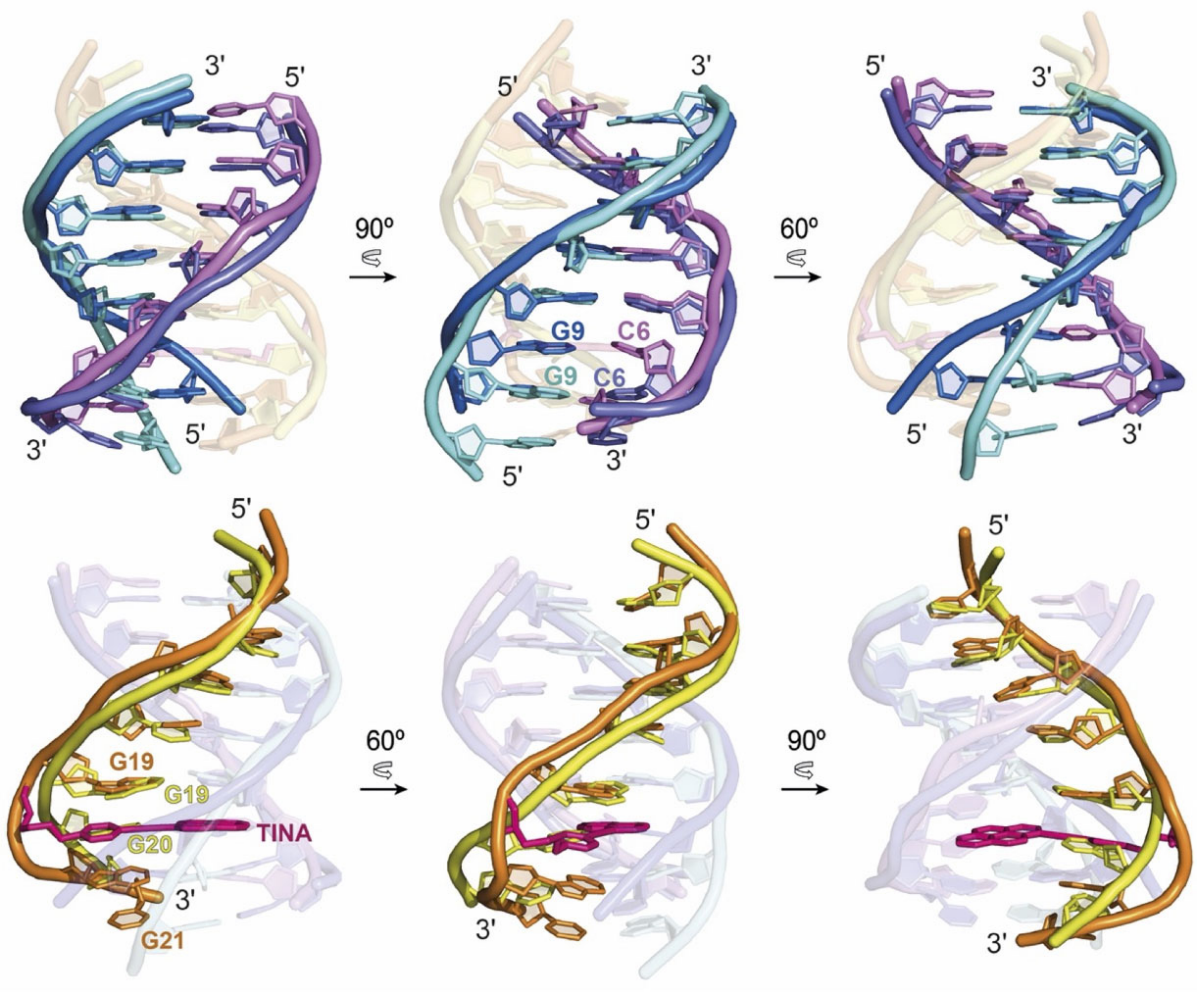
Structural alignment of TTa and the homologous region of the unmodified triplex PDB ID 134D (45) (UT134D). Top. The Watson–Crick strands (*slate blue*: TTa; *violet*: UT134D) and the purine strands (*light blue*: TTa; *dark blue*: UT134D) are mostly overlapped along the segment from T1:A14 to C5:G10. C6 and G9 of TTa are moved and tilted to make a wider gap between C5:G10 and C6:G9 base pairs, facilitating the intercalation of the pyrene moiety of TINA. Bottom. The Hoogsteen strands of TTa (*orange*) and UT134D (*yellow*) show a poor alignment. The bases from both strands show different tilt and the backbone of TTa is placed at longer distance to the purine strand. Bases flanking TINA (G19 and G20) are more distant to each other with respect to the homologous G19 and G20 in UT134D. G20 is tilted towards 3′-end to increasing the space for TINA intercalation. The conjugation of TINA results in a distortion of the backbone of TTa which shows as a bulge between G19 and G20.

A comparison between the structure of TTa and the structure of the unmodified triplex reported by Radhakrishnan and Patel (45) (PDB ID: 134D) is shown in Figure 5. Although TINA intercalation provokes large local distortions in the surrounding nucleobases, the overall triplex structure is mainly unaffected. The pyrene ring is sandwiched between the Watson–Crick pairs, with a large stacking surface with the guanine nucleobases. In addition, the phenyl ring stacks on top of the 5′-neighbouring guanine within its own strand. These stabilizing interactions, primarily resulting from highly favourable stacking, compensate for the local distortions at the intercalation site and are responsible for the significant stabilization caused by TINA. This explains the enhanced stability of antiparallel triplexes formed by TFOs containing TINA monomers between two guanines (purines), instead of between a purine and a pyrimidine (35). This sequence design also reduces the ability of G-rich strands to form G-quadruplexes (35,36) which contributes to the shift of triplex–G-quadruplex equilibrium towards triplex formation. It is also interesting to notice that TINA fits very well with the overall size of Pyrimidine:Purine*Purine (Y:R*R) triad. Although a significant bulge is observed in the Hoogsteen strand, no large distortions occur in the purine and pyrimidine strands backbone. Since TINA is probably more difficult to accommodate between the smaller Y:R*Y triads, this may explain the enhanced stabilization of TINA in antiparallel vs parallel triplex (35). The good fitting of TINA between Y:R*R triads suggests that only a few modifications in its chemical design such as using polyaromatic platforms slightly larger than phenyl (68) and pyrene may improve TINA-induced antiparallel triplex stability.

The use of modified TFOs with improved stability inside cells and higher affinity for the target duplex (24,69–73) is further expanding the potential of triplex formation as a tool for therapeutic and biotechnological applications. Particularly, *para*-TINA modification favours the formation of more stable triplexes without compromising the specificity. Indeed, the thermal stability of parallel triplexes formed by TINA-modified TFOs decreases more than 11.5ºC when the resulting triplex has a single mismatch (38). Similarly, for TINA-modified TFOs forming antiparallel triplexes, the presence of a mismatch next to TINA completely disrupts triplex formation (36,37). Moreover, the fluorescence properties of TINA can be used in probes for detection of DNA and RNA specific sequences (74–79). The structural details unveiled in this study help rationalize the different effects of TINA insertion when it occurs in the middle of purine-rich strands of TFOs and in G4-forming sequences. Insertion in quadruplex G-tracts usually results in lower thermal stability or the formation of higher-order structures through TINA-TINA interactions (64,80), making this modification a very useful tool to modulate G-rich sequence folding and to overcome the main limitations of triplex formation and visualization *in vitro* and *in vivo*.

The high-resolution structural data presented here will greatly contribute to a more rational and efficient design of modified oligonucleotides aimed to be used as antigenic agents and DNA-based biotechnological devices. These results are of interest for further design of more efficient modulators of hybridization properties of oligonucleotides allowing the formation and stabilization of complexes with genomic DNA for therapeutic and diagnostic applications.

## Supplementary Material

gkae028_Supplemental_File

## Data Availability

The data underlying this article are available in the Protein Data Bank (PDB) at https://www.rcsb.org/, and can be accessed with ID 8PWR. All other data are available in the article or Supplementary information. Raw data will be shared on request to the corresponding/first authors.

## Notes

Present address: Israel Serrano-Chacón, Institute for Research in Biomedicine (IRB Barcelona), The Barcelona Institute of Science and Technology (BIST), 08028, Barcelona, Spain.

Present address: Osman Doluca, Faculty of Engineering, Department of Biochemical Engineering. Izmir University of Economics, Izmir 35330, Turkey.
