# Supplementary material for: Understanding intercalative modulation of G-rich sequence folding: solution structure of a TINA-conjugated antiparallel DNA triplex
Source: Nucleic Acids Res. 2024 Jan 28;52(5):2686–97. doi: 10.1093/nar/gkae028 (PMC10954471; doi:10.1093/nar/gkae028)
Supplement: gkae028_Supplemental_File [file gkae028_supplemental_file.pdf]

Supplementary Data

Understanding intercalative modulation of G-rich sequence folding: solution structure of a TINA-conjugated antiparallel DNA triplex

Miguel Garavís[1], Patrick J. B. Edwards[2], Israel Serrano-Chacón[1], Osman Doluca[2], Vyacheslav V. Filichev[2,*] and Carlos González[1,*]

[1] Instituto de Química Física 'Blas Cabrera', (IQF-CSIC), Madrid, 28006, Spain
[2] School of Natural Sciences, Massey University, Palmerston North, 4412, New Zealand

* To whom correspondence should be addressed.
Carlos González. phone: +34 915619400; Email: cgonzalez@iqfr.csic.es

Vyacheslav V. Filichev. phone: +64 69517659; Email: v.filichev@massey.ac.nz

Outline:

A. Supplementary Methods and References.

- Determination of thermodynamic parameters from UV-melting profiles p2
- References p2

B. Supplementary Figures

Figure S1. UV melting curves, CD spectra and plots for thermodynamic analysis. p3
Figure S2. NOESY and TOCSY spectra of TTa in D_2O at 5ºC. p4
Figure S3. Assignment of TINA protons (NOESY and TOCSY in D_2O). p5
Figure S4. Imino region of the NOESY spectrum of TTa in H_2O at 5ºC. p6
Figure S5. Imino region (G9 and G10) of the NOESY spectrum of TTa in H_2O at 5ºC. p7
Figure S6. Imino region (H2' – imino) of the NOESY spectrum of TTa in H_2O at 5ºC. p8
Figure S7. Chemical shift differences between UT and TTa protons. p9
Figure S8. Ensemble of the solution structures of TTa. p10
Figure S9. RMSD evolution along unbiased MD simulation. p11
Figure S10. Selected helical parameters of the solution structure of TTa. p12

C. Supplementary Tables

Table S1. Assignment list of DNA protons of TTa. p13
Table S2. Assignment list of aromatic TINA protons. p13
Table S3. DNA – TINA cross-peaks. p14
Table S4. Assignment list of UT protons. p15
Table S5. Highest chemical shift differences between TTa and UT protons. p16
Table S6. Experimental constraints and calculation statistics of TTa. p17
Table S7. Average pseudorotation parameters of TTa. p18
Table S8. Average dihedral angles and order parameters of TTa. p19
Table S9. Atomic partial charges of TINA using HF and B3LYP methods. p20

A. Supplementary Methods and References

Determination of thermodynamic parameters from UV-melting.

To analyze thermodynamic parameters of unimolecular triplexes, melting profiles obtained from UV melting experiment (Fig S1 A) were converted into a fraction folded (ϴ) vs temperature representation (Fig. S1 C and D) by choosing lower and upper baselines:

$$\Theta_T = (L0_T - C_T) / (L0_T - L1_T) \quad \text{(Equation 1)}$$

Where C_T is the UV signal at 256 nm at a given temperature, $L0_T$ and $L1_T$ correspond to the baseline values of the unfolded and folded species, respectively. ϴ is a number between 0 and 1: $\Theta = 0$ for $T >> T_m$, $\Theta = 1$ for $T << T_m$, and $\Theta = 0.5$ for $T = T_m$.

By definition, the free Gibbs enthalpy may be written as:

$$\Delta G^0 = -RT \ln(K_a) = \Delta H^0 - T \times \Delta S^0 \quad \text{(Equation 2)}$$

Where R = 8.3145 J/(K·mol), T is the temperature in Kelvin, ΔH^0 is the standard enthalpy of the reaction, and ΔS^0 is the standard entropy, assuming that $\Delta Cp = 0$[46]

Equation 2 can be deduced as:

$$\ln(K_a) = -\Delta H^0 / R \times (1/T) + \Delta S^0 / R \quad \text{(Equation 3)}$$

Therefore, the following step required a van't Hoff plot of the natural logarithm of the affinity constant ($\ln(K_a)$) as a function of the reciprocal of the temperature (1/T in K^{-1})[S1].

For unimolecular equilibrium A ⇔ B:

$$K_a = [B] / [A] \quad \text{(Equation 4)}$$

When A and B is present at equilibrium then:

$$K_a = \Theta / (1 - \Theta) \quad \text{(Equation 5)}$$

where ϴ is Θ_T at each temperature.

It should be noted that the analysis should be restricted between the temperature range for which $0.15 < \Theta < 0.85$ as it is relatively difficult to evaluate the affinity constant when almost all or almost none of the molecules are associated[S2].

Following the calculations described above, Fig. S1 C and D was converted into Fig. S1 E and F, respectively. The van't Hoff relation ($\ln(K_a)$ vs. 1/T) should give a straight line (linear regression), with a slope of $-\Delta H^0/R$ and Y-axis intercept of $\Delta S^0/R$ thus providing thermodynamic parameters for all complexes listed in the Table in Fig S1.

References:

[S1] M. Mills, P. B. Arimondo, L. Lacroix, T. Garestier, C. Hélène, H. Klump, J.-L. Mergny, *J. Mol. Biol.* **1999**, *291*, 1035-1054.

[S2] J. D. Puglisi, I. Tinoco, Jr., *Methods Enzymol.* **1989**, *180*, 304-325.

B. Supplementary Figures.

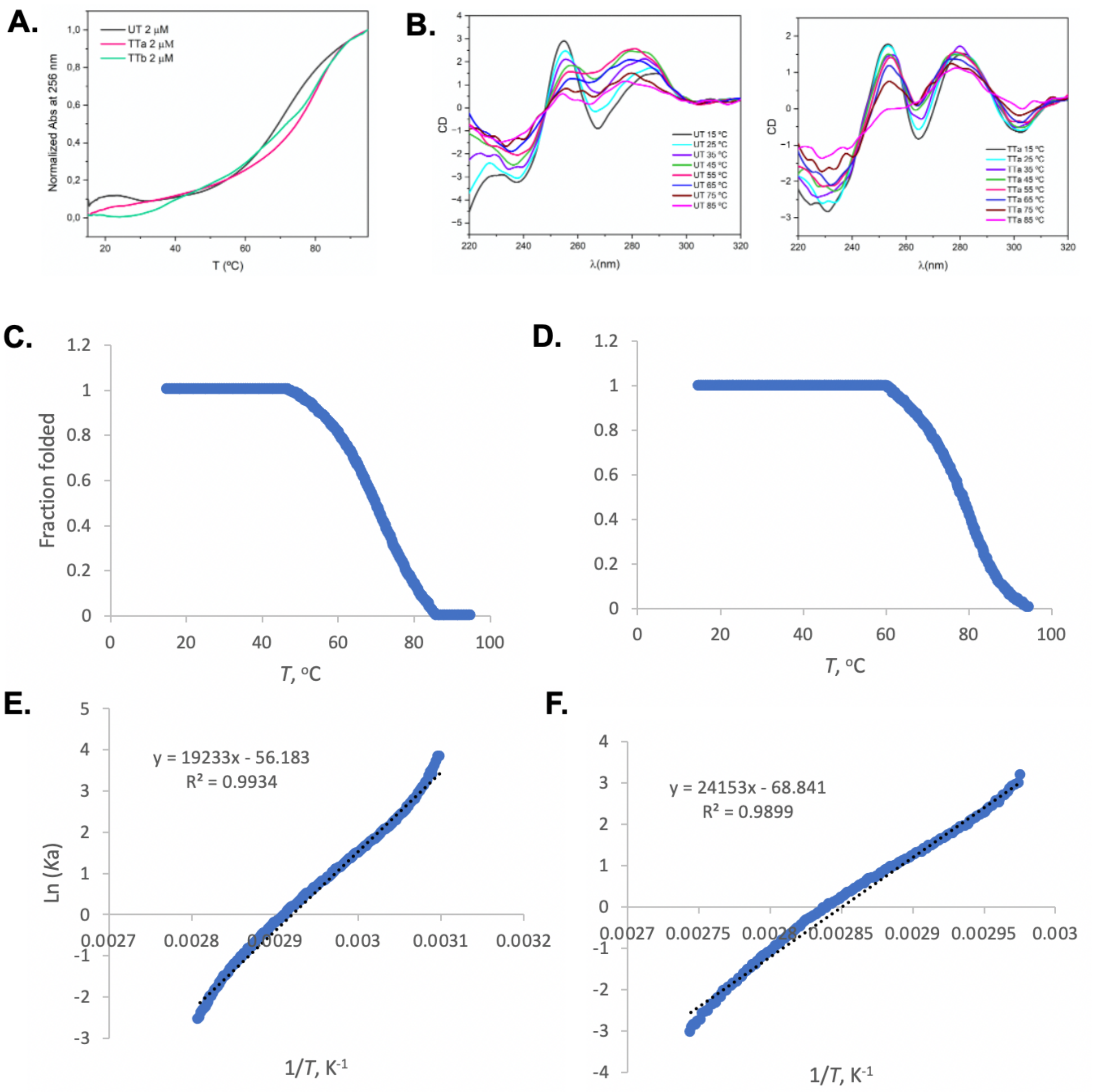

The T_M [°C] and thermodynamic data at 298 K for the antiparallel triplexes, taken from UV melting curves.				
	T_M [°C]	Δ*H* (kJ/mol)	*T*Δ*S* (kJ/mol)	ΔG_{298} (kJ/mol)
**UT**	69	-160 (1)	-139.3 (0.9)	-20.7 (0.2)
**TTa**	78	-201 (2)	-170.6 (1.5)	-30.4 (0.4)

Figure S1. (A) UV melting curves of UT (black), TTa (red) and TTb (green). Estimated T_M for UT and TTa are 69 ºC and 78 ºC, respectively. T_M of TTb could not be fitted to a single transition. (B) CD spectra of UT (left) and TTa (right) at different temperatures, ranging from 15 to 85 ºC. Buffer conditions: 25 mM sodium phosphate and 100 mM NaCl, pH 7. DNA concentration was 2 μM for UV and 20 μM for CD experiments. Plots of (C and D) fraction folded (Θ) vs temperature and (E and F) the natural logarithm of the affinity constant (ln(K_a)) as a function of the reciprocal of the temperature (1/T in K^{-1}) for unmodified (UT, left panels) and TINA-modified triplexes (TTa, right panels). (Bottom) The table provides T_M and thermodynamic data of triplex formation (see section A in the Supplementary Data).

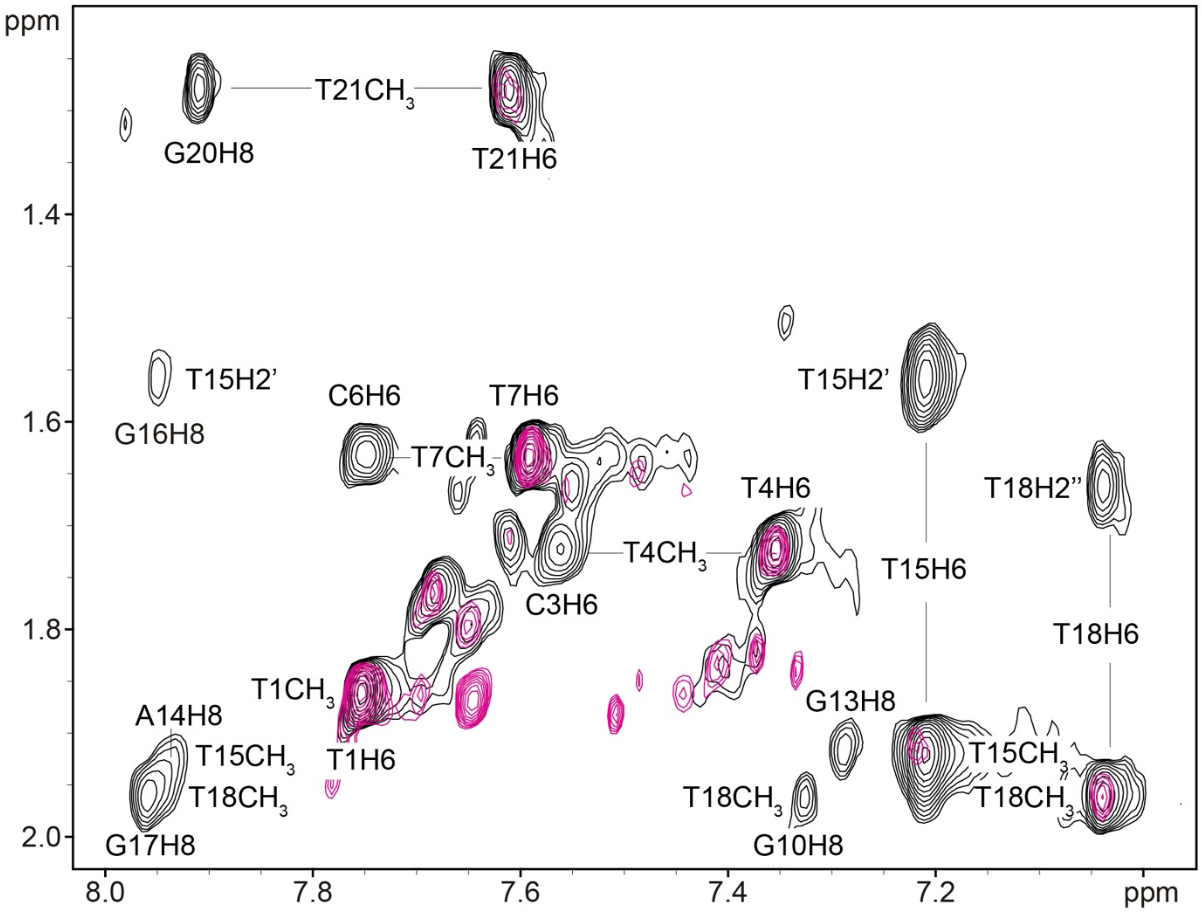

Figure S2. NOESY (black) and overlaid TOCSY (pink) spectra of TTa in D_2O at 5ºC showing intra- and inter-residual NOEs between methyl and aromatic protons. Methyl protons of thymines in C-T steps of the first strand ($T4CH_3$ and $T7CH_3$) show cross-peaks with aromatic protons of the previous cytosine (C3H6 and C6H6). Aromatic protons of guanines in G-A steps of the second strand (G10H8 and G13H8) show cross-peaks with methyl protons of thymines of the third strand ($T18CH_3$ and $T15CH_3$). Right-handed helicity of third strand is confirmed by cross-peaks between aromatic protons of guanines in steps G-T (G17H8 and G20H8) with methyl protons of next guanine in the sequence ($T18CH_3$ and $T21CH_3$).

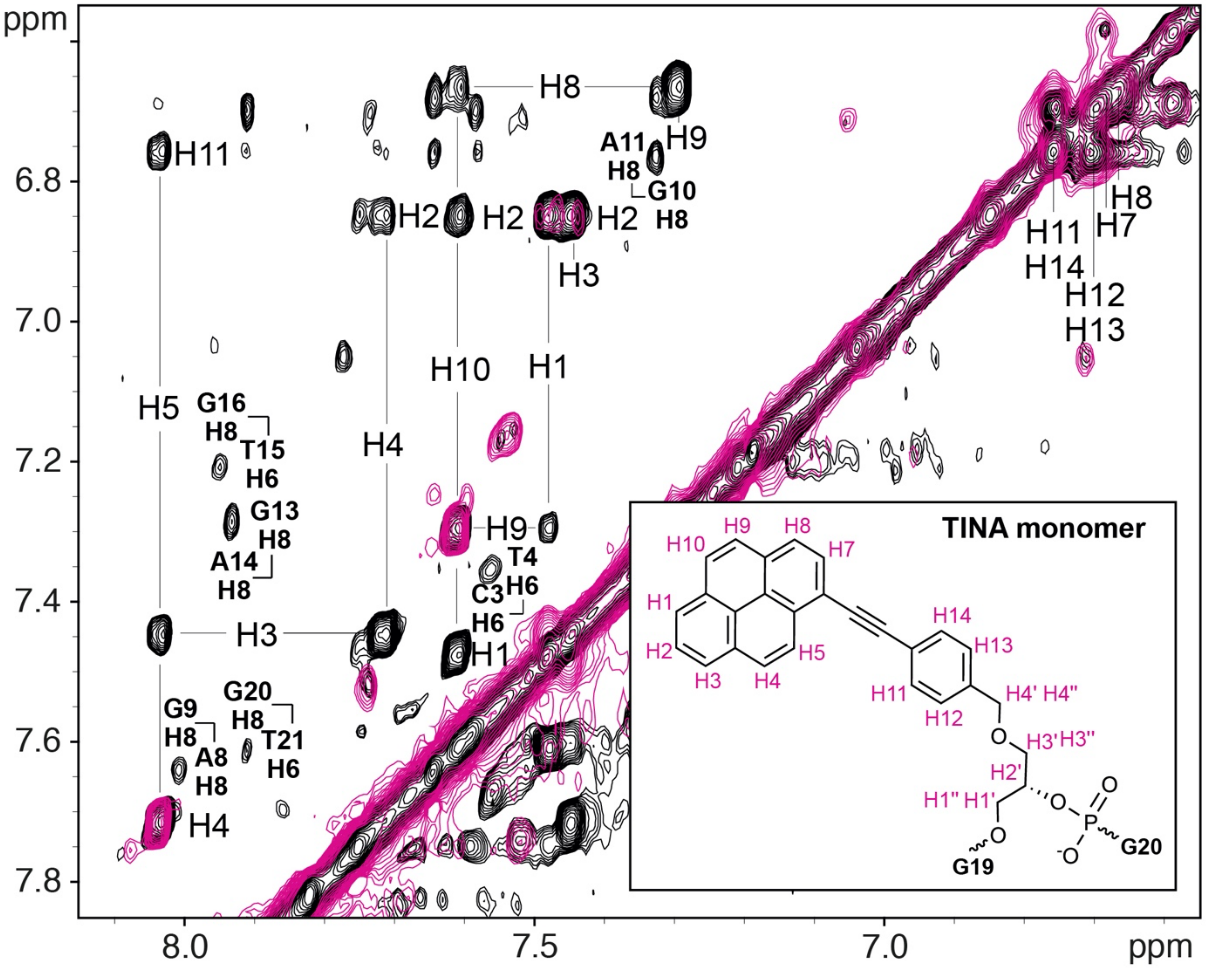

Figure S3. Aromatic region of the NOESY (black) and TOCSY (pink) spectra of TTa in D_2O at 5ºC showing aromatic-aromatic protons in TINA (regular font) and in DNA (bold font). Note TOCSY signals between protons separated by three bonds.

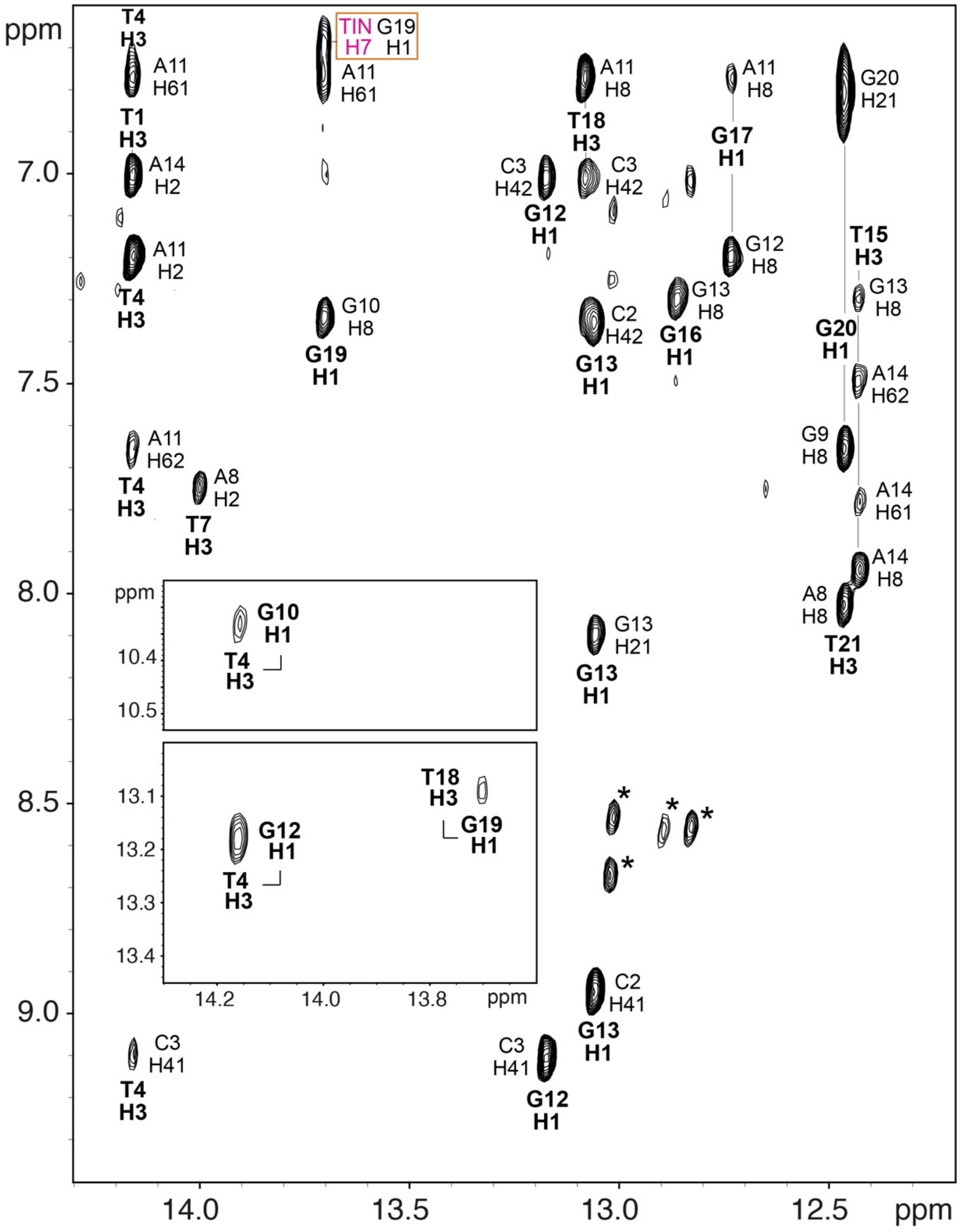

Figure S4. Imino region of the NOESY spectrum of TTa in H_2O at 5ºC showing cross-peaks characteristic of the formation of C:G*G and T:A*T triads. Labels in bold identify imino signals. The NOEs along each vertical lines correspond to cross-peaks between the corresponding imino signal and the protons labelled aside of the peak. The insets show imino-imino cross-peaks observed between consecutive residues in the structure. Signals marked with an asterisk correspond to residual alternative structure in equilibrium with the triplex.

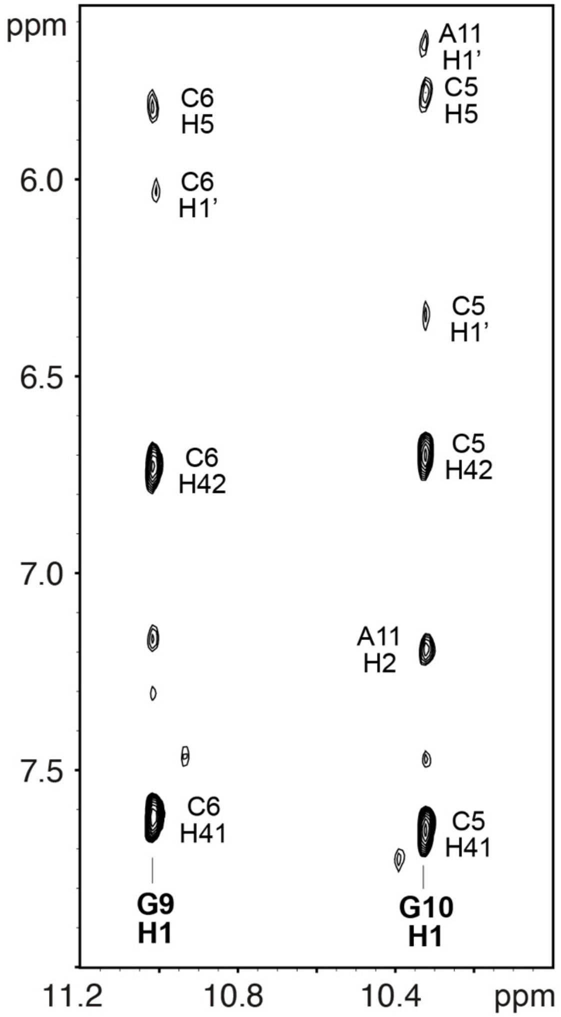

Figure S5. Region of the NOESY spectrum of TTa in H_2O at 5ºC showing resonances of G9 and G10 imino protons (bold) and their cross-peaks with protons of their Watson-Crick base-paired cytosines. G10H1 also shows weak NOEs with H2 and H1' of its flanking adenine A11. H41 are the designated as the amino protons involved in the Watson-Crick base pair while H42 are the unbound amino protons.

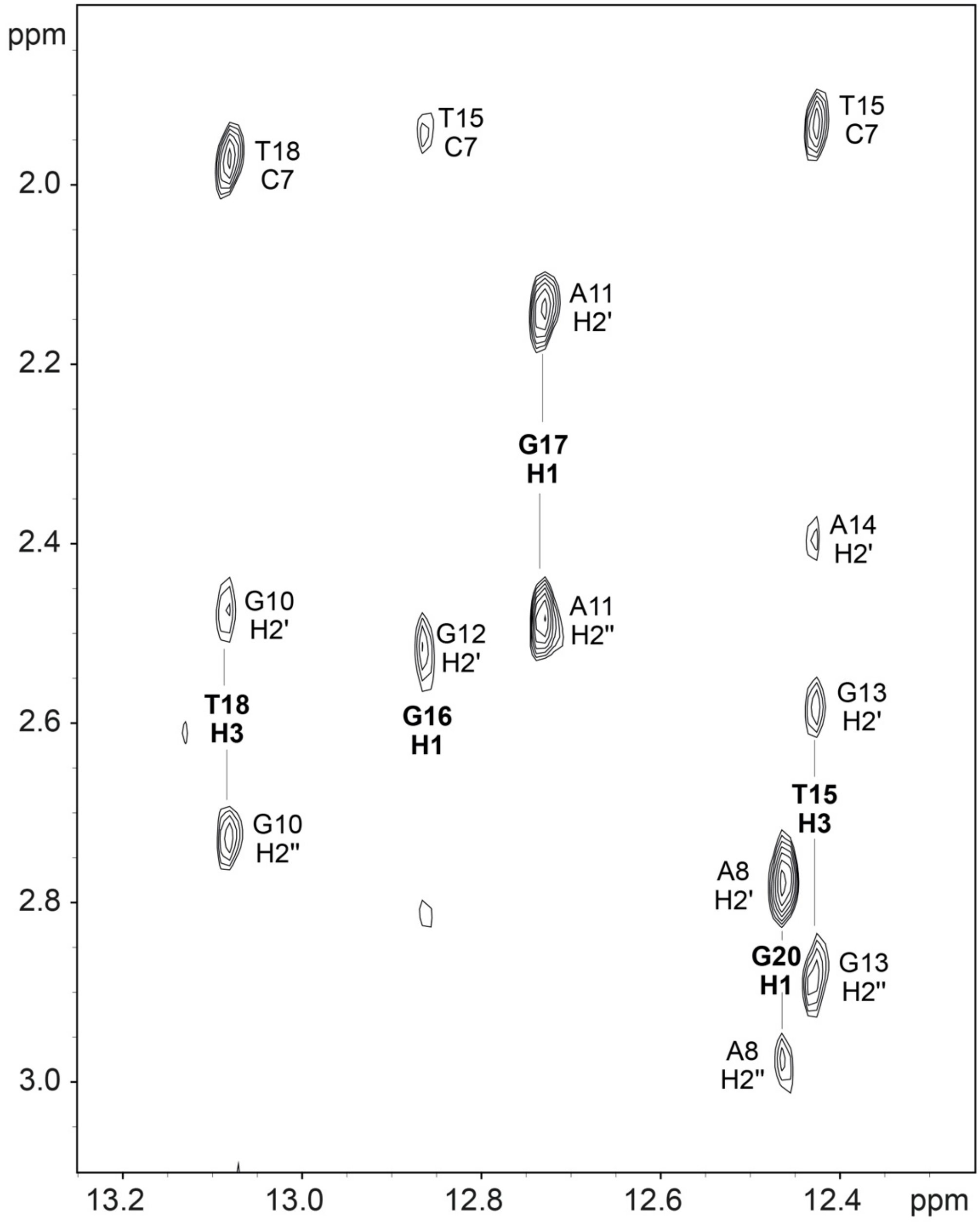

Figure S6. Imino region of the NOESY spectrum of TTa in H_2O at 5ºC showing cross-peaks between imino protons of residues of the Hoogsteen strand (bold) and sugar protons of residues preceding their base-paired nucleotide in the purine-rich strand.

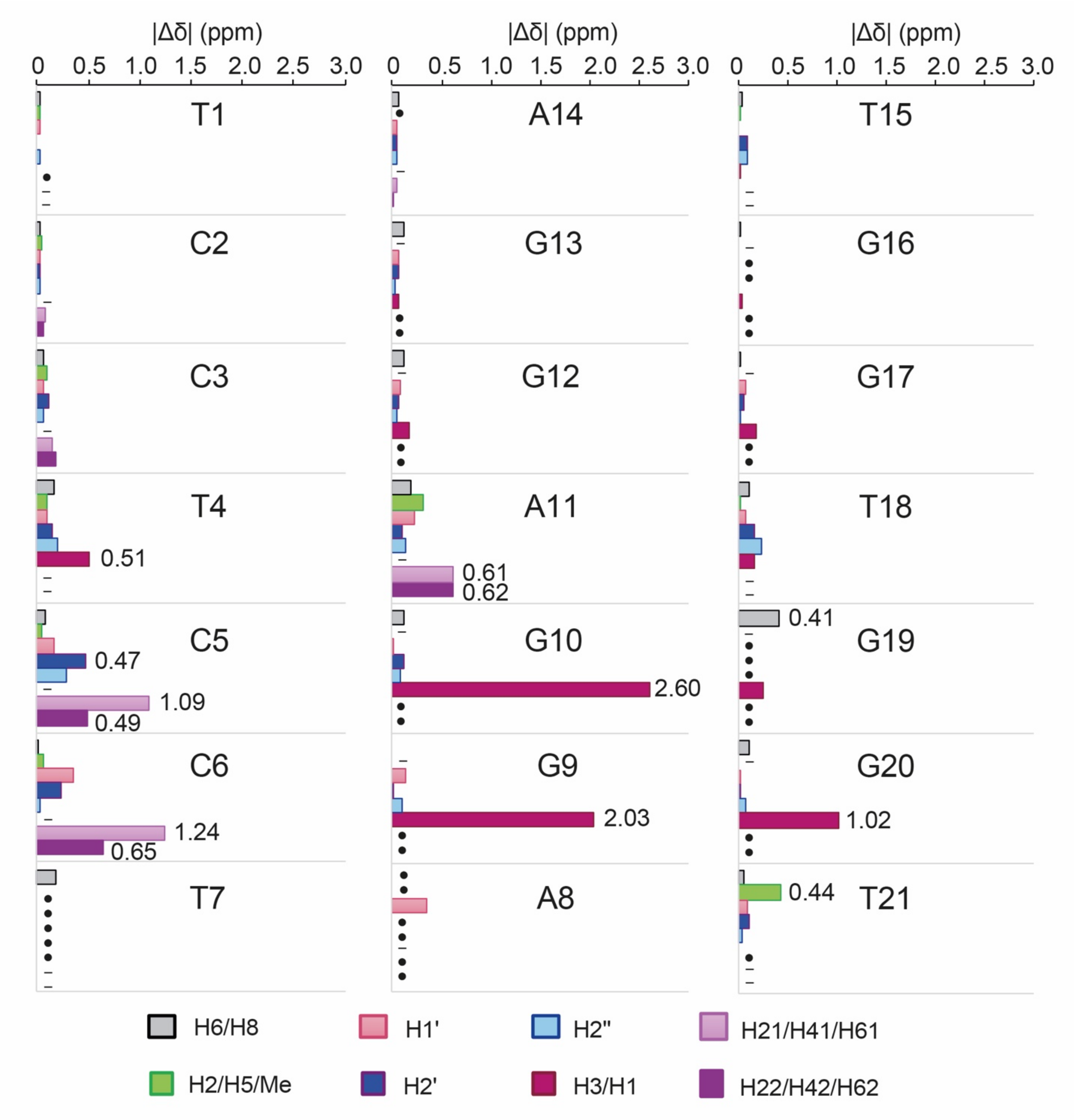

Figure S7. Bar chart showing the chemical shift differences between equivalent protons of TTa and UT (in absolute values). The highest values are indicated beside the bars. Dots indicate that the corresponding difference was not calculated because the proton was not assigned for UT or TTa. The short lines indicate non existing proton in that particular residue.

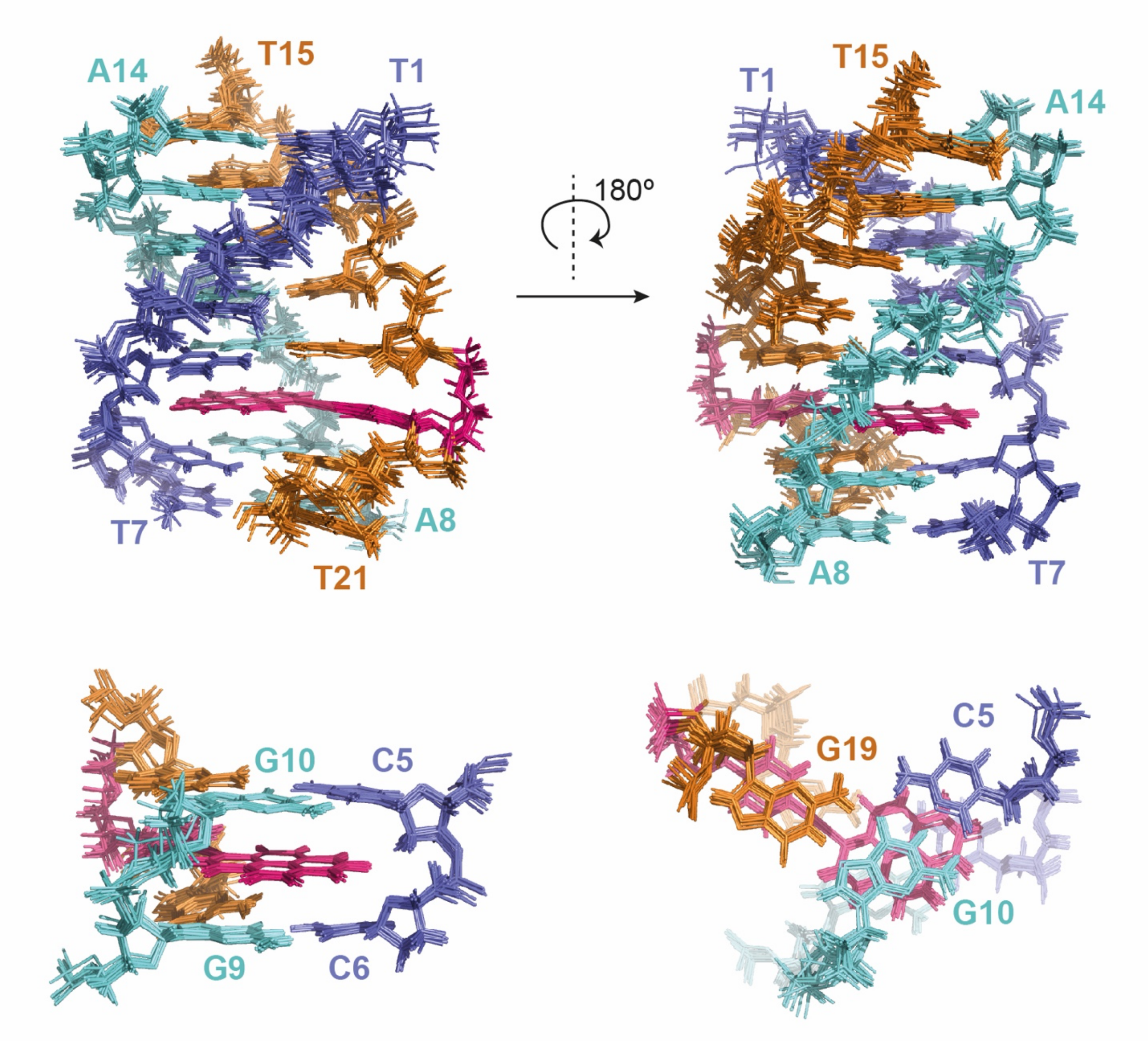

Figure S8. Two views of the ensemble of ten structures of TTa determined from NMR data (PDB 8PWR). Top) General view. Bottom) Detailed of TINA and neighbouring residues. Colour code as in Figure 4 in the main text.

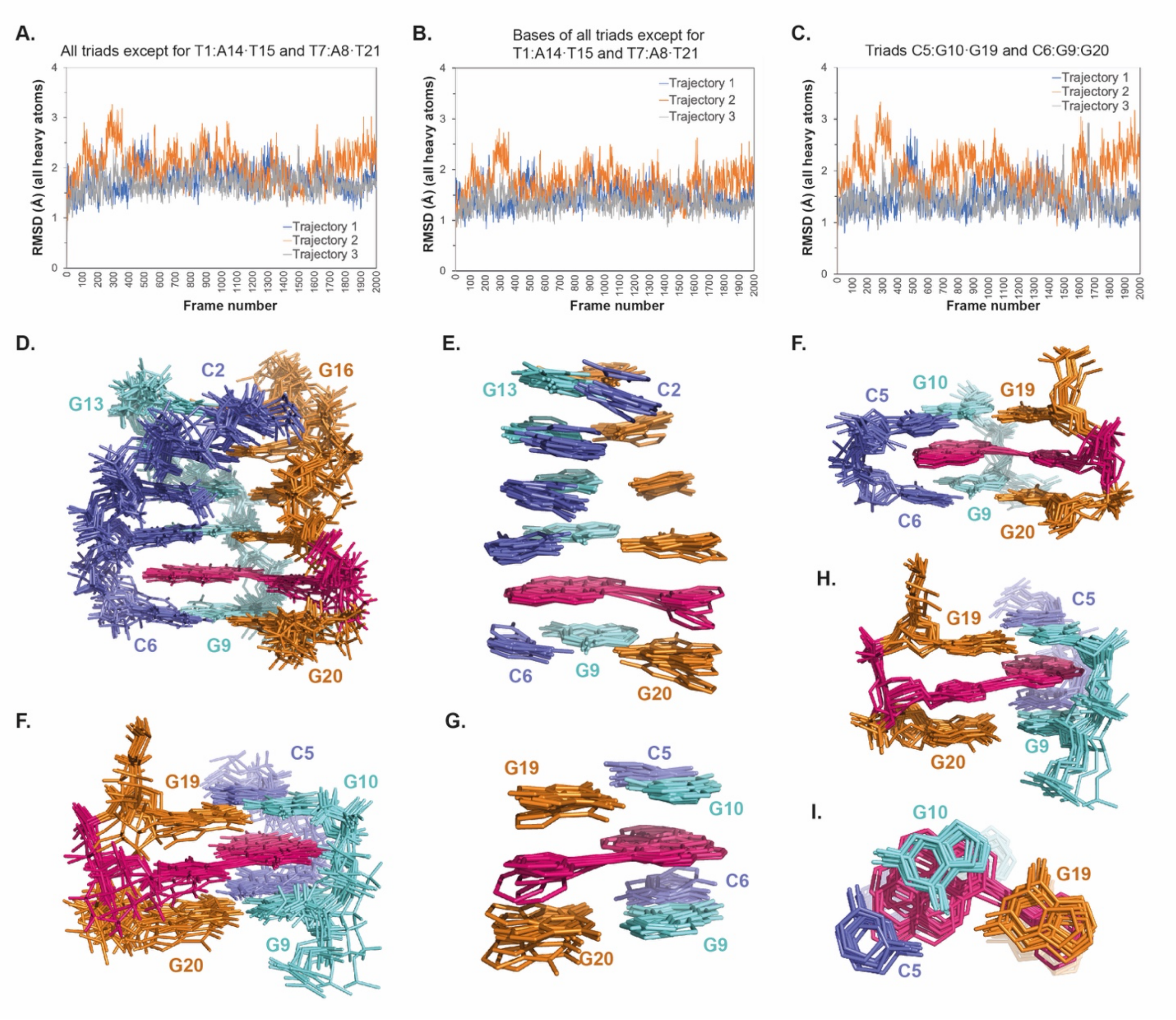

Figure S9. Plots showing the RMSD variation along 3 replicas (trajectories 1, 2 and 3) of unbiased molecular dynamics of 100 ns duration, considering (left) all heavy atoms except the terminal triads (T1:A14·T15 and T7:A8·T21); (middle) base atoms except the terminal triads, and (right) TINA atoms and the neighbouring triads. The RMSDs are calculated with respect to the seed TTa structure (frame 0). Different views of the superposition of snapshots along the trajectories, showing the overall structure (D), the nucleobases (E), and details of the TINA interaction site (F..I).

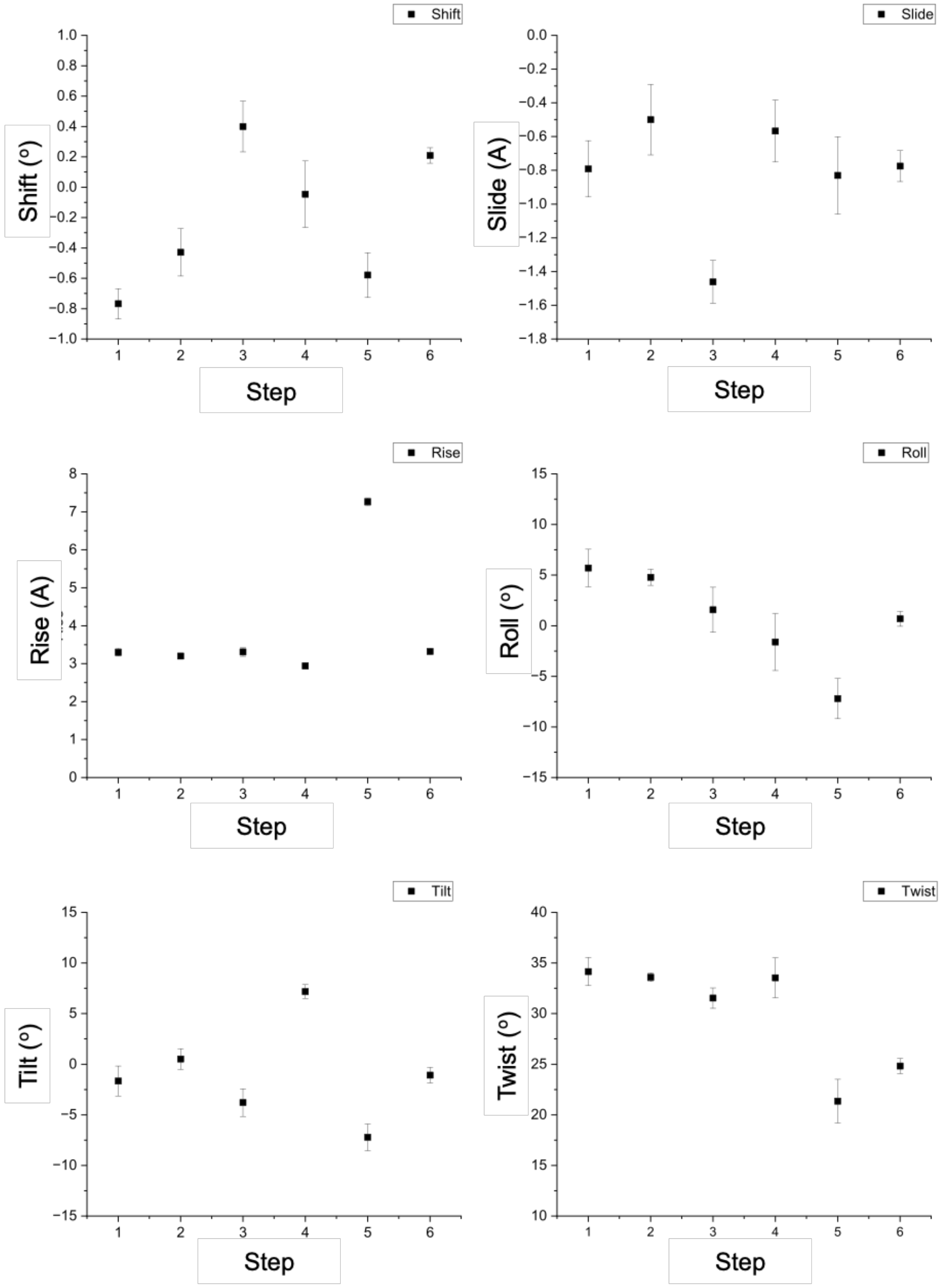

Figure S10. Selected helical parameters of the solution structure of TTa. Plotted values and error bars correspond to the average and mean square deviations of the helical parameters calculated for each of the ten structures.

C. Supplementary Tables

Table S1. Chemical shifts of TTa protons (ppm).
n.a. Not assigned

Res.	**H6/H8**	**H2/H5/Me**	**H1'**	**H2'**	**H2''**	**H3'**	**H4'**	**H3/H1**	**H21/H41/H61**	**H22/H42/H62**
**T1**	7.75	1.86	6.13	2.36	2.66	4.79	4.17	14.16	-	-
**C2**	7.68	5.74	6.19	2.31	2.66	4.78	4.27	-	8.95	7.36
**C3**	7.56	5.50	6.05	2.24	2.7	n.a.	4.26	-	9.11	7.01
**T4**	7.35	1.72	5.84	2.49	2.06	n.a.	4.02	14.16	-	-
**C5**	7.6	5.77	6.35	2.59	2.82	5.13	4.20	-	7.65	6.7
**C6**	7.75	5.82	6.03	2.2	2.48	4.71	4.33	-	7.62	6.73
**T7**	7.59	1.63	6.32	2.34	2.54	4.91	4.23	14.00	-	-
**A8**	8.01	7.75	5.95	2.78	2.97	4.91	4.25	-	n.a.	n.a.
**G9**	7.64	-	5.76	2.69	2.77	5.1	n.a.	11.02	n.a.	n.a.
**G10**	7.33	-	5.96	2.47	2.73	n.a.	4.29	10.32	n.a.	n.a
**A11**	6.76	7.20	5.66	2.13	2.47	4.56	4.33	-	7.66	6.77
**G12**	7.19	-	5.88	2.5	2.8	4.89	4.41	13.17	n.a.	n.a.
**G13**	7.29	-	5.83	2.57	2.88	5.07	4.34	13.06	n.a.	n.a.
**A14**	7.93	7	6.21	2.39	2.57	4.94	4.40	-	7.78	7.49
**T15**	7.21	1.92	5.52	1.56	1.95	4.64	3.87	12.43	-	-
**G16**	7.95	-	n.a.	n.a.	n.a.	n.a.	n.a.	12.86	n.a.	n.a.
**G17**	7.96	-	5.69	2.27	2.87	4.84	n.a.	12.73	n.a.	n.a.
**T18**	7.04	1.96	6.07	1.08	1.66	4.85	4.08	13.08	-	-
**G19**	7.58	-	5.62	2.58	2.64	n.a.	4.30	13.7	n.a.	n.a.
**G20**	7.91	-	6.16	2.77	2.77	4.72	4.46	12.46	6.80	n.a.
**T21**	7.61	1.28	6.43	2.21	2.29	4.62	4.28	12.46	-	-

Table S2. Assignment list of aromatic TINA protons.

Residue	H1	H2	H3	H4	H5	H7	H8	H9	H10	H11	H12	H13	H14
TINA	7.48	6.85	7.45	7.71	8.03	6.69	6.67	7.30	7.61	6.76	6.70	6.70	6.76

**Table S3**: DNA – TINA cross-peaks		
	Number	Cross-peaks (NOEs)
TINA – DNA First strand	18	C5H2' – TINA H1
		C5H2' – TINA H2
		C5H2' – TINA H3
		C5H2'' – TINA H1
		C5H2'' – TINA H2
		C5H2'' – TINA H3
		C5H5 – TINA H3
		C5H1' – TINA H1
		C5H1' – TINA H2
		C5H42 – TINA H3
		C5H42 – TINA H4
		C6H5 – TINA H2
		C6H5 – TINA H3
		C6H6 – TINA H2
		C6H1' – TINA H1
		C6H1' – TINA H2
		C6H1' – TINA H10
		C6H42 – TINA H4
TINA – DNA Second strand	12	G9H2' – TINA H7
		G9H2' – TINA H8
		G9H2'' – TINA H7
		G9H2'' – TINA H8
		G9H2'' – TINA H14
		G9H3' – TINA H8
		G9H1' – TINA H7
		G9H1' – TINA H8
		G9H8 – TINA H7
		G9H8 – TINA H14
		G10H4' – TINA H8
		G10H8 – TINA H7
TINA – DNA Third strand	15	G19H2' – TINA H12
		G19H2'' – TINA H12
		G19H1' – TINA H11
		G19H1' – TINA H12
		G19H8 – TINA H13
		G19H8 – TINA H14
		G20H2' – TINA H12/13
		G20H2'' – TINA H12/13
		G20H4' – TINA H12
		G20H5' – TINA H12
		G20H5'' – TINA H12
		G20H1' – TINA H11
		G20H1' – TINA H12
		G20H8 – TINA H12
		G20H8 – TINA H11
TOTAL	45	-

**Table S4.** Chemical shifts of UT protons (ppm).<br>n.a. Not assigned										
Res.	**H6/H8**	**H2/H5/Me**	**H1'**	**H2'**	**H2"**	**H3'**	**H4'**	**H3/H1**	**H21/H41/H61**	**H22/H42/H62**
**T1**	7.77	1.89	6.16	2.36	2.69	4.80	4.18	n.a.	-	-
**C2**	7.70	5.79	6.21	2.34	2.69	4.80	4.28	-	9.03	7.42
**C3**	7.63	5.60	6.12	2.36	2.77	4.84	4.31	-	9.26	7.20
**T4**	7.51	1.81	5.93	2.63	2.26	4.88	4.15	14.67	-	-
**C5**	7.68	5.72	6.19	2.12	2.54	4.89	n.a.	-	8.74	7.19
**C6**	7.76	5.88	6.38	2.44	2.51	4.92	4.24	-	8.86	7.38
**T7**	7.78	n.a.	n.a.	n.a.	n.a.	n.a.	n.a.	n.a.	-	-
**A8**	n.a.	n.a.	5.61	n.a.	n.a.	n.a.	n.a.	-	n.a.	n.a.
**G9**	7.64	-	5.89	2.68	2.87	n.a.	n.a.	13.05	8.50	7.22
**G10**	7.22	-	5.97	2.35	2.82	4.36	4.17	12.92	8.57	7.13
**A11**	6.95	7.52	5.88	2.23	2.6	4.51	n.a.	-	7.05	7.39
**G12**	7.31	-	5.96	2.56	2.85	n.a.	4.44	13.34	8.60	7.09
**G13**	7.40	-	5.90	2.64	2.91	4.95	4.40	13.13	7.83	7.47
**A14**	7.99	n.a.	6.26	2.44	2.61	n.a.	4.42	-	7.74	7.50
**T15**	7.26	1.94	5.52	1.65	2.04	4.67	3.90	12.45	-	-
**G16**	7.98	-	5.52	2.50	2.55	4.75	4.05	12.91	n.a.	n.a.
**G17**	7.99	-	5.77	2.33	2.89	n.a.	4.37	12.92	n.a.	n.a.
**T18**	7.15	1.97	6.14	1.25	1.90	n.a.	4.12	13.25	-	-
**G19**	7.99	-	n.a.	n.a.	n.a.	n.a.	n.a.	13.44	n.a.	n.a.
**G20**	8.02	-	6.19	2.79	2.85	4.76	4.47	13.48	n.a.	n.a.
**T21**	7.55	1.72	6.34	2.33	2.33	4.61	n.a.	n.a.	-	-

Table S5. Chemical shifts differences between UT and TTa protons (ppm).
n.o. Not obtained

Res.	H6/H8	H2/H5/Me	H1'	H2'	H2''	H3'	H4'	H3/H1	H21/H41/H61	H22/H42/H62
**T1**	0.02	0.03	0.03	0	0.03	0.01	0.01	n.o.	n.o.	n.o.
**C2**	0.02	0.05	0.02	0.03	0.03	0.02	0.01	n.o.	0.08	0.06
**C3**	0.07	0.10	0.07	0.12	0.07	n.o.	0.05	n.o.	0.15	0.19
**T4**	0.16	0.09	0.09	0.14	0.20	n.o.	0.13	0.51	n.o.	n.o.
**C5**	0.08	0.05	0.16	0.47	0.28	0.24	n.o.	n.o.	1.09	0.49
**C6**	0.01	0.06	0.35	0.24	0.03	0.21	0.09	n.o.	1.24	0.65
**T7**	0.19	n.o.	n.o.	n.o.	n.o.	n.o.	n.o.	n.o.	n.o.	n.o.
**A8**	n.o.	n.o.	0.34	n.o.	n.o.	n.o.	n.o.	n.o.	n.o.	n.o.
**G9**	0	n.o.	0.13	0.01	0.10	n.o.	n.o.	2.03	n.o.	n.o.
**G10**	0.11	n.o.	0.01	0.12	0.09	n.o.	0.12	2.60	n.o.	n.o.
**A11**	0.19	0.32	0.22	0.10	0.13	0.05	n.o.	n.o.	0.61	0.62
**G12**	0.12	n.o.	0.08	0.06	0.05	n.o.	0.03	0.17	n.o.	n.o.
**G13**	0.11	n.o.	0.07	0.07	0.03	0.12	0.06	0.07	n.o.	n.o.
**A14**	0.06	n.o.	0.05	0.05	0.04	n.o.	0.02	n.o.	0.04	0.01
**T15**	0.05	0.02	0	0.09	0.09	0.03	0.03	0.02	n.o.	n.o.
**G16**	0.03	n.o.	n.o.	n.o.	n.o.	n.o.	n.o.	0.05	n.o.	n.o.
**G17**	0.03	n.o.	0.08	0.06	0.02	n.o.	n.o.	0.19	n.o.	n.o.
**T18**	0.11	0.01	0.07	0.17	0.24	n.o.	0.04	0.17	n.o.	n.o.
**G19**	0.41	n.o.	n.o.	n.o.	n.o.	n.o.	n.o.	0.26	n.o.	n.o.
**G20**	0.11	n.o.	0.03	0.02	0.08	0.04	0.01	1.02	n.o.	n.o.
**T21**	0.06	0.44	0.09	0.12	0.04	0.01	n.o.	n.o.	n.o.	n.o.

**Table S6**: Experimental constraints and calculation statistics of TTa.		
Experimental distance constraints		
Total number	152	
Intra-residue	57	
Sequential	95	
Range > 1	49	
Inter TINA-Triplex	42	
RMSD (Å)		
All bases	0.3 ± 0.1 Å	
Backbone	0.6 ± 0.2 Å	
All heavy atoms	0.6 ± 0.2 Å	
Residual violations	Average	Range
Sum of violation (Å)	4.41	4.08 – 4.59
Max. violation (Å)	0.33	0.32 – 0.34
NOE energy# (kcal/mol)	20.4	19.5 – 21.1
Total energy (kcal/mol)	-914	-943 – -865

K_{NOE} = 20 kcal/(mol.Å^2)

**Table S7.** Average pseudorotation parameters of TTa			
**Residue**	**Phase**	**Amplitude**	**Puckering**
T1	176	42	C2'-endo
C2	163	36	C2'-endo
C3	159	37	C2'-endo
T4	145	35	C2'-endo
C5	61	34	C4'-exo
C6	155	42	C2'-endo
T7	159	39	C2'-endo
A8	186	36	C2'-endo
G9	145	46	C2'-endo
G10	174	41	C2'-endo
A11	170	38	C2'-endo
G12	165	38	C2'-endo
G13	141	39	C2'-endo
A14	145	44	C2'-endo
T15	50	36	C4'-exo
G16	177	38	C2'-endo
G17	166	36	C2'-endo
T18	52	24	C4'-exo
G19	270	30	C2'/C3'-endo
G20	132	45	C1'-exo
T21	157	35	C2'-endo

Table S8. Average dihedral angles (Aver.) and order parameters (O.P.) of TTa.

Residue	α		β		γ		δ		ε		χ	
	Aver.	O.P.	Aver.	O.P.	Aver.	O.P.	Aver.	O.P.	Aver.	O.P.	Aver.	O.P.
T1	-	-	-	-	-80	1.0	157	1.0	-177	1.0	-113	1.0
C2	-77	1.0	-170	1.0	55	1.0	142	1.0	-174	1.0	-113	1.0
C3	-77	1.0	-176	1.0	55	1.0	140	1.0	173	1.0	-110	1.0
T4	-69	1.0	-167	1.0	52	1.0	131	1.0	-162	1.0	-124	1.0
C5	-85	1.0	166	1.0	59	1.0	89	1.0	179	1.0	-130	1.0
C6	-53	1.0	-172	1.0	75	1.0	143	1.0	-148	1.0	-110	1.0
T7	-93	1.0	64	1.0	178	1.0	148	1.0	-	-	-162	1.0
A8	-	-	-	-	-125	0.5	153	1.0	-167	1.0	-108	1.0
G9	-81	1.0	174	1.0	47	1.0	137	1.0	-156	1.0	-100	1.0
G10	43	0.4	-159	0.5	-70	0.9	153	1.0	176	1.0	-92	1.0
A11	-83	0.9	151	0.5	97	0.5	148	1.0	-163	1.0	-125	0.9
G12	27	0.3	-176	0.9	-62	0.3	138	0.9	-173	1.0	-117	1.0
G13	-73	1.0	174	1.0	54	1.0	127	1.0	-173	1.0	-118	1.0
A14	-72	1.0	177	1.0	51	1.0	138	1.0	-	-	-114	1.0
T15	-	-	-	-	171	1.0	84	1.0	-178	1.0	-105	1.0
G16	-65	1.0	168	1.0	68	1.0	155	1.0	-97	1.0	-86	1.0
G17	-80	1.0	148	1.0	50	1.0	142	1.0	-180	1.0	-109	1.0
T18	-78	1.0	-161	1.0	50	1.0	93	1.0	-167	1.0	-98	1.0
G19	-75	1.0	-176	1.0	56	1.0	100	1.0	-154	1.0	-86	1.0
G20	-60	1.0	171	1.0	69	1.0	126	1.0	-179	1.0	-132	1.0
T21	-69	1.0	-174	1.0	56	1.0	141	1.0	-	-	-108	1.0

Table S9. Charges of TINA atoms.			
	HF/ 6-31g(d)	B3LYP/ 6-31G(d,p)	Difference
**P**	1.1659	1.1659	0
**OP1**	-0.7761	-0.7761	0
**OP2**	-0.7761	-0.7761	0
**C4'**	0.193014	0.130951	0.062063
**C3'**	-0.096314	-0.105324	0.00901
**O3'**	-0.5232	-0.5232	0
**C1'**	0.254637	0.315926	-0.061289
**C2'**	0.179759	0.278278	-0.098519
**C1**	-0.272431	-0.206703	-0.065728
**O1'**	-0.388352	-0.327289	-0.061063
**C2**	-0.104267	-0.094845	-0.009422
**C3**	-0.278146	-0.211528	-0.066618
**C3a**	0.186413	0.136009	0.050404
**C4**	-0.262776	-0.216031	-0.046745
**C5**	-0.183778	-0.14461	-0.039168
**C5a**	0.111186	0.062634	0.048552
**C6**	0.044226	0.076214	-0.031988
**C7**	-0.151379	-0.142692	-0.008687
**C8**	-0.292267	-0.218773	-0.073494
**C8a**	0.186113	0.13109	0.055023
**C9**	-0.225184	-0.182316	-0.042868
**C10**	-0.236474	-0.195254	-0.04122
**C10a**	0.168246	0.121584	0.046662
**C10b**	0.009244	0.024803	-0.015559
**C10c**	-0.053651	-0.020788	-0.032863
**C11**	-0.259655	-0.200484	-0.059171
**C12**	-0.091882	-0.097505	0.005623
**C12a**	0.089543	0.100573	-0.01103
**C13**	-0.265413	-0.222942	-0.042471
**C14**	-0.176478	-0.136733	-0.039745
**C14a**	0.243444	0.190307	0.053137
**C15**	-0.21957	-0.169902	-0.049668
**C16**	-0.070452	-0.088229	0.017777
**H1**	0.164607	0.128322	0.036285
**H2**	0.144716	0.119442	0.025274
**H3**	0.168233	0.131201	0.037032
**H4**	0.164758	0.136733	0.028025
**H5**	0.160624	0.13147	0.029154
**H7**	0.16504	0.137007	0.028033
**H8**	0.173976	0.136336	0.03764
**H9**	0.155041	0.128314	0.026727
**H10**	0.161081	0.132962	0.028119
**H11**	0.183787	0.145626	0.038161
**H12**	0.130196	0.123561	0.006635
**H13**	0.145622	0.11548	0.030142
**H14**	0.163673	0.132212	0.031461
**H1'**	-0.007996	-0.050421	0.042425
**H3''**	0.090239	0.072756	0.017483
**H3'**	0.090239	0.072756	0.017483
**H4''**	0.036758	0.042926	-0.006168
**H4'**	0.036758	0.042926	-0.006168
**H2'**	0.048188	-0.010709	0.058897
**O5'**	-0.4954	-0.4954	0
**H1''**	-0.007996	-0.050421	0.042425

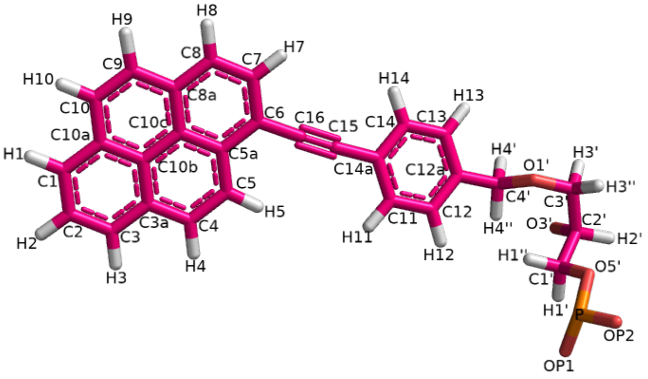

Values for charges of TINA atoms obtained using HF/6-31g(d) and B3LYP/6-31G(d,p) protocols. Fourth column shows the difference between the values using the two different parametrization methods.
